# 3D‐hUMSCs Exosomes Ameliorate Vitiligo by Simultaneously Potentiating Treg Cells‐Mediated Immunosuppression and Suppressing Oxidative Stress‐Induced Melanocyte Damage

**DOI:** 10.1002/advs.202404064

**Published:** 2024-06-17

**Authors:** Qi Wang, Weinan Guo, Liaoran Niu, Yuqi Zhou, Zeqian Wang, Jianru Chen, Jiaxi Chen, Jingjing Ma, Jia Zhang, Zhaoting Jiang, Bo Wang, Zhe Zhang, Chunying Li, Zhe Jian

**Affiliations:** ^1^ Department of Dermatology Xijing Hospital Fourth Military Medical University Xi'an Shaanxi 710032 China; ^2^ Department of Digestive Surgery Xijing Hospital Fourth Military Medical University Xi'an Shaanxi 710032 China

**Keywords:** 3D culture, autoimmunity, exosomes, human umbilical cord mesenchymal stem cells, vitiligo

## Abstract

Vitiligo is an autoimmune disease characterized by epidermal melanocyte destruction, with abnormal autoimmune responses and excessive oxidative stress as two cardinal mechanisms. Human umbilical mesenchymal stem cells‐derived exosomes (hUMSCs‐Exos) are regarded as promising therapeutic choice for autoimmune diseases due to potent immunosuppressive and anti‐oxidative properties, which can be potentiated under 3D cell culture condition. Nevertheless, whether exosomes derived from 3D spheroids of hUMSCs (3D‐Exos) exhibit considerable therapeutic effect on vitiligo and the underlying mechanism remain elusive. In this study, systemic administration of 3D‐Exos showed a remarkable effect in treating mice with vitiligo, as revealed by ameliorated skin depigmentation, less CD8^+^T cells infiltration, and expanded Treg cells in skin, and 3D‐Exos exerted a better effect than 2D‐Exos. Mechanistically, 3D‐Exos can prominently facilitate the expansion of Treg cells in vitiligo lesion and suppress H_2_O_2_‐induced melanocytes apoptosis. Forward miRNA profile analysis and molecular experiments have demonstrated that miR‐132‐3p and miR‐125b‐5p enriched in 3D‐Exos greatly contributed to these biological effects by targeting *Sirt1* and *Bak1* respectively. In aggregate, 3D‐Exos can efficiently ameliorate vitiligo by simultaneously potentiating Treg cells‐mediated immunosuppression and suppressing oxidative stress‐induced melanocyte damage via the delivery of miR‐132‐3p and miR‐125b‐5p. The employment of 3D‐Exos will be a promising treament for vitiligo.

## Graphical abstract

HUMSCs‐Exos can significantly suppress vitiligo mouse depigmentation in vivo. Mechanistically, the 3D‐Exos enriched with miR‐132‐3p and miR‐125b‐5p can induce Treg cells differentiation and suppress oxidative stress‐induced apoptosis by targeting *Sirt1* and *Bak1* respectively, thereby protecting melanocytes from oxidative and immune destruction, and ultimately alleviating the progression of vitiligo.

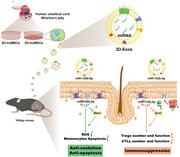

## 1 Introduction

Vitiligo is a common skin disease characterized by melanocytes destruction in the epidermis or mucous membrane and the resultant formation of white spots and patches, leading to patients’ disfiguration and anxiety that has brought a heavy burden to society and family^[^ ^1^ ^]^ To date, numerous mechanisms have been identified to contribute to vitiligo pathogenesis, among which autoimmunity and oxidative stress are two cardinal events mediating melanocyte destruction.^[^ ^2^ ^]^ Based on previous investigations conducted by our group and others, it has been well documented that oxidative stress acts as the initial event of melanocyte damage, whereas abnormal autoimmunity is the predominant contributor to ultimate melanocyte destruction.^[^ ^1^, ^2^, ^3^ ^]^ However, there is still a lack of effective therapeutic approach simultaneously targeting autoimmunity and oxidative stress in vitiligo treatment, which proposes the need of further development of alternative treatment method.

Human mesenchymal stem cells (hMSCs) are versatile stromal cells with multi‐lineage differentiation ability, anti‐inflammatory and immunosuppressive capacity, as well as anti‐oxidative effect, which can be derived from several sources including the umbilical cord, fat tissue, and bone marrow. These characteristics make them an ideal choice for regeneration and immunomodulation, especially as a promising stem cell‐based therapy for a series of autoimmune diseases,^[^ ^4^ ^]^ such as psoriasis, systemic lupus erythematosus, Sjogren's syndrome, multiple sclerosis, type 1 diabetes, rheumatoid arthritis, and vitiligo. However, multiple disadvantages including immune rejection,^[^ ^5^ ^]^ undirected cell differentiation^[^ ^6^ ^]^ and tumorigenicity have posed obstacles in the path of applying hMSCs‐based therapies in clinical applications.^[^ ^7^ ^]^ Therefore, it is important to develop alternative strategies that can improve the advantage of hUMSCs properties.

Recent accumulating evidence has revealed that the therapeutic efficacy of hUMSCs is mainly attributed to their paracrine secretion of extracellular vesicles (EVs).^[^ ^8^ ^]^ Exosome, a major class of EVs with the diameter ranging from 30–150 nm, is the most well‐characterized and widely‐investigated one in paracrine secretion, playing critical roles in both physiological and pathological processes.^[^ ^9^ ^]^ By transferring bioactive molecules like DNA, mRNAs, miRNAs, proteins, and lipids from donor to recipient cells, exosomes serve as cardinal mediator of intercellular communication.^[^ ^10^ ^]^ In contrast to transplanted exogenous hMSCs, exosomes derived from their parent hMSCs have been documented to elicit considerable therapeutic effects,^[^ ^11^ ^]^ and being regarded as a cell‐free therapy vehicle due to higher safety and lower immunogenicity.^[^ ^8b^ ^]^ Of note, recent studies have demonstrated that hUMSCs‐Exos have shown remarkable efficacy on alleviating inflammation, autoimmune response and oxidative injury,^[^ ^12^ ^]^ indicating its great potential as highly promising nanotherapeutic agents for the treatment of multiple oxidative stress and autoimmune‐related diseases.^[^ ^13^ ^]^

Though hUMSCs‐derived exosomes have shown explicit treatment potential, the efficacy is limited when hUMSCs are cultured under conventional 2D culture condition.^[^ ^14^ ^]^ Therefore, pre‐conditioning strategies like 3D aggregation culture of MSCs have been developed to facilitate paracrine secretion of exosomes and improve therapeutic quality,^[^ ^15^ ^]^ endowing 3D‐cultured MSCs‐Exos (3D‐Exos) with a higher yield^[^ ^16^ ^]^ and better therapeutic effects by transferring specific cargoes.^[^ ^12^, ^14^ ^]^ In particular, 3D culture systems have been shown to increase the total production of exosomes by over 19‐fold compared to conventional 2D culture.^[^ ^17^ ^]^ Moreover, exosomes produced from 3D cultures of MSCs showed higher yield and improved internalization activity,^[^ ^16c^ ^]^ significantly potentiating the therapeutic effect on Alzheimer's disease,^[^ ^12a^ ^]^ periodontitis, and colitis in pre‐clinical models.^[^ ^18^ ^]^ Nevertheless, whether hUMSCs‐Exos exerts therapeutic effect on vitiligo, and whether 3D‐Exos exhibits better therapeutic effects than conventional 2D culture conditions, have not been investigated.

In the present study, we first investigated the biological characteristics of hUMSCs‐Exos produced in 2D and 3D culture conditions. Subsequently, through the establishment of melanoma‐Treg‐induced vitiligo mouse model, the therapeutic effect of 3D‐Exos on vitiligo was tested and compared with that of 2D‐Exos in vivo. Forward functional studies in vitro were conducted to figure out the role of 3D‐Exos in Treg cells‐mediated immunosuppression and oxidative stress‐induced melanocyte damage. Ultimately, detailed mechanisms underlying the function of 3D‐Exos were demonstrated, with particular emphasis on miRNAs transferred by 3D‐Exos and their corresponding targets in Treg cells and melanocytes, respectively.

## 2 Results

### 2.1 Establishment and Characterization of hUMSCs in 2D and 3D Culture

**Figure 1:**
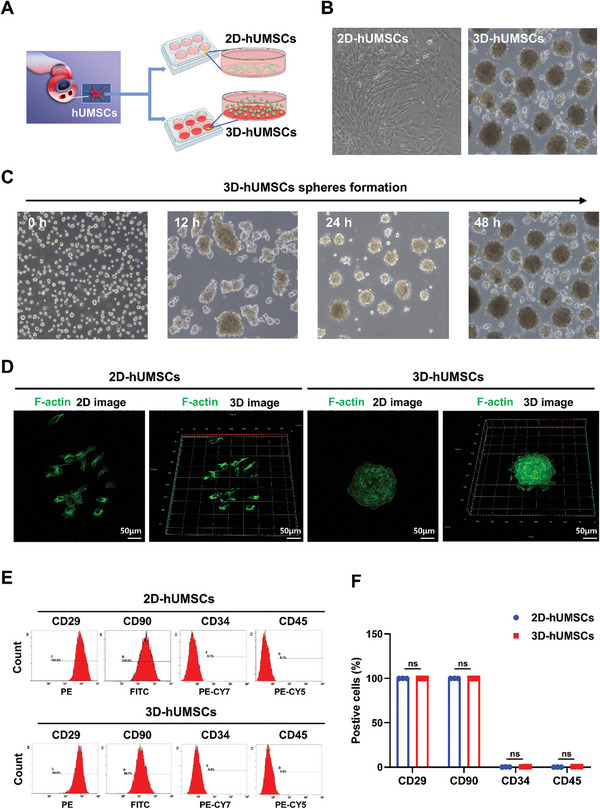
The establishment of 2D and 3D culture systems and the identification of hUMSCs. A,B) Schematic diagrams and light microscopes were used to observe hUMSCs cultured under 2D and 3D conditions. C) The light microscope revealed that hUMSCs gradually formed spheres over time under 3D culture conditions. D) Immunofluorescence staining analysis was performed on F‐actin in hUMSCs grown in 2D and 3D cultures. E,F) Surface markers of hUMSCs were analyzed by flow cytometry (*n* = 3 per group). Error bars represented mean ± standard deviation (SD). ns indicates not statistically significant.

hUMSCs were successfully isolated from umbilical cord wharton's jelly of the healthy donors, and the schematic diagram of conventional 2D and 3D culture systems was shown in **Figure** **1**A. Optical microscopy illustrated that hUMSCs in 2D culture system presented the homogeneous population of spindle fibroblast‐like cells (Figure 1B). However, hUMSCs in 3D culture system spontaneously aggregated and changed into compact multicellular spheroids after suspension culture for 48 h (Figure 1B,C). 2D immunofluorescence images revealed that the morphology of hUMSCs cultured in 2D monolayer was single and dispersed through staining of actin filaments. In contrast, 3D reconstruction from z‐stack confocal images showed that hUMSCs cultured in 3D suspension were interconnected and compacted within the diameters of 100–200 µm (Figure 1D). Flow cytometry analysis revealed that both groups of hUMSCs were highly positive (>99%) for MSCs surface markers including CD29 and CD90, but negative (<1%) for hematopoietic stem cells surface markers CD34 and CD45 (Figure 1E). Interestingly, no significant difference was observed in the hUMSCs surface markers expression between 2D‐hUMSCs and 3D‐hUMSCs, suggesting that phenotype of hUMSCs was not changed under 3D‐culture condition (Figure 1F).

### 2.2 Isolation and Characterization of 2D‐Exos and 3D‐Exos

**Figure 2:**
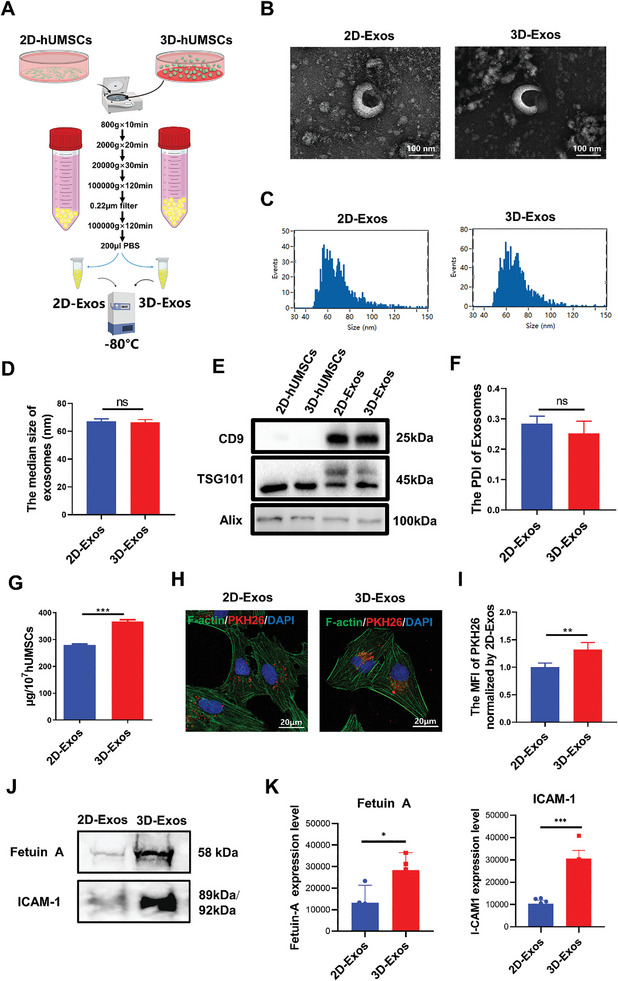
Identification and uptake of exosomes derived from supernatant of 2D and 3Dcultured hUMSCs. A) Schematic representation of exosome isolation from hUMSCs supernatant by ultracentrifugation. B) The morphology of exosome was observed by TEM. C,D) The particle size distribution of hUMSCs‐Exos was measured using a nanoflow cytometer (*n* = 3 per group). E) Exosome markers CD9, TSG101, and Alix were detected by western blot. F) The PDI of 2D‐Exos and 3D‐Exos was measured using Zetasizer Nano‐S90 (*n* = 3 per group). G) Exosome protein concentration in the 2D‐Exos and 3D‐Exos groups was examined using the BCA assay (*n* = 3 per group). H,I) Uptake of the PKH26‐labeled 2D‐Exos and 3D‐Exos by PIG3V cells was observed by fluorescence microscopy, and mean fluorescence intensity was statistically evaluated (*n* = 5 per group). J,K) Representative western blot analysis and quantification of Fetuin A and ICAM‐1proteins expression in 2D‐Exos and 3D‐Exos groups. Each lane represented an independent biological replicate. Error bars represented mean ± SD. Statistical significance was denoted as ^*^ *p* < 0.05, ^**^ *p* < 0.01, and ^***^ *p* < 0.001 by t test (D,F,G1 I,K); ns indicates not statistically significant.

Exosomes were isolated from the serum‐free supernatants of hUMSCs cultured in both 2D and 3D systems through a standard method of serial ultracentrifugation, and the exosome pellets were reconstituted in PBS and stored at −80 °C before utilization (**Figure** **2**A). To understand the impact of the 3D cellular spheroids on the physical and biochemical properties of EVs, we performed transmission electron microscopy (TEM), nanoflow cytometer (NanoFCM), and western blot analysis of 2D‐Exos and 3D‐Exos. TEM revealed that the vesicles displayed ≈100 nm spherical particles with typical sphere‐shaped bilayer membrane structure, similar to the recognized characteristics of exosomes (Figure 2B). A high‐resolution NanoFCM showed that the size of vesicles range from 30 to 150 nm in diameter, and the median diameters of 2D‐Exos and 3D‐Exos were 67.24 nm ± 1.41 nm and 66.51 nm ± 1.56 nm respectively, which met the typical criteria for exosomes (Figure 2C,D). Western blot analysis showed that the exosome surface proteins cluster of differentiation 9 (CD9), tumor susceptibility gene 101 (TSG101), and ALG‐2‐interacting protein X (Alix) were expressed in both 2D‐Exos and 3D‐Exos (Figure 2E). The polydispersity index (PDI) of 2D‐Exos and 3D‐Exos were also examined and displayed to be comparable (Figure 2F). These results indicated that there were no differences in the morphology, size, PDI, or exosomal markers between these two groups, and 2D‐Exos and 3D‐Exos could be successfully isolated according to the above procedures.

Thereafter, in order to investigate the yields of exosomes, supernatants (at a total volume of 50 mL) were collected from 1 × 107 hUMSCs in 2D and 3D culture systems respectively, and the bicinchoninic acid (BCA) assay was utilized to determine the yields of 2D‐Exos and 3D‐Exos. Compared with 2D‐Exos, the protein abundance was significantly increased in 3D‐Exos (Figure 2G), supporting that the yields of hUMSCs‐Exos from 3D culture system are higher than those from 2D culture system. To examine whether exosomes derived from 3D or 2D culture conditions were taken up differentially by vitiligo melanocyte cell line PIG3V, we incubated cultured PIG3V cells with PKH26‐labeled exosomes and observed that exosomes in both groups were internalized by PIG3V cells after 24 h as indicated by confocal laser scanning microscopy (CLSM) (Figure 2H). However, the number of exosomes taken up by PIG3V cells was significantly higher in 3D‐Exos group compared with 2D‐Exos group (Figure 2I), suggesting that 3D‐Exos were more easily taken up by PIG3V cells. To explore the underlying mechanisms, we further investigated the expression of exosome internalization‐associated proteins. BCA method was used to quantify exosomes and equal amounts of exosomes were used for western blot experiments. The results revealed a significant upregulation in the expression levels of alpha‐2‐HS‐glycoprotein (Fetuin A) and intercellular adhesion molecule‐1 (ICAM‐1) proteins in exosomes exposed to 3D culture condition compared to 2D culture condition, suggesting that the increased expression of internalization‐associated proteins in 3D‐Exos may contribute to the enhanced internalization by PIG3V cells (Figure 2J, K).

### 2.3 3D‐Exos Treatment Significantly Ameliorated Depigmentation in Mouse Model of Vitiligo

**Figure 3:**
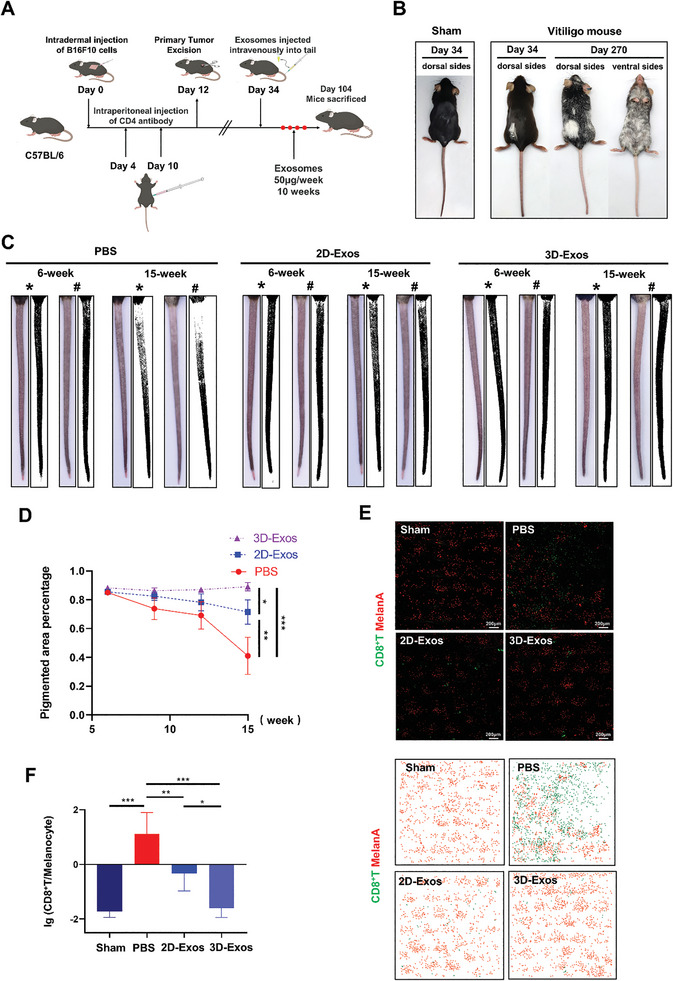
Establishment of vitiligo mouse model and tail vein administration of hUMSCs‐Exos. A) Schematic presentation of vitiligo mouse model establishment. B) Representative pictures of wild‐type mice and vitiligo mice at different stages. C) Representative tail images and ImageJ analysis of mice in PBS (*n* = 3 or 4 per group), 2D‐Exos (*n* = 4 per group), or 3D‐Exos (*n* = 4 per group) group at weeks 6 and 15 (* dorsal, # ventral). D) The tail pigmentation percentages of mice in the three groups in 10 consecutive weeks (from week 6 to week 15) were calculated using Image J software (*n* = 3 or 4 per group). E) Whole‐mount immunofluorescent staining images of CD8^+^T cells (green) and melanocytes (red), and corresponding heatmaps of CD8^+^T cells (green) and melanocytes (red) in the tail epidermis of vitiligo mice (*n* = 3 or 4 per group). F) Statistical analysis of lg value of the number of CD8^+^T/melanocyte in whole‐mount immunofluorescent staining. All sham mice were only treated with PBS. Error bars represented mean ± SD. Statistical significance was denoted as ^*^ *p* < 0.05, ^**^ *p* < 0.01, and ^***^ *p* < 0.001 by one‐way analysis of variance (ANOVA) and Tukey's test (D,F).

To evaluate the in vivo effect of hUMSCs‐Exos in treating vitiligo, and to compare the therapeutic efficacy of 2D‐Exos and 3D‐Exos, a melanoma‐Treg‐induced vitiligo mouse model that recapitulated the pathological characteristic of human patients with vitiligo was established as previously described (**Figure** **3**A).^[^ ^19^ ^]^ Flow cytometry analysis revealed nearly complete elimination of CD4^+^ T cells in peripheral blood of the mice after injection of CD4 antibody (Figure S1A, Supporting Information). On day 34 after the vitiligo induction procedure, CD8^+^T cells infiltration could be observed in the tail epidermis by CLSM (Figure S1B, Supporting Information), and dorsal skin hair follicles close to the surgical removal sites started to show depigmentation in vitiligo mouse (Figure 3B). Then the depigmented hair follicles expanded and eventually rendered the most parts of dorsal and ventral hair coats depigmented at ≈Day 270. Meanwhile, the depigmentation in tail skin was initially patchy and then progressed to eventually cover the entire skin (Figure 3B). As is known, epidermal depigmentation is the defining feature of vitiligo. Of note, melanocytes in the dorsal skin of mice are predominantly found within hair follicles, rather than the epidermis. However, in the skin of the mouse tail, melanocytes are uniquely situated in both the hair follicle and the epidermis, a distribution that more closely resembles the pattern observed in human skin.^[^ ^19^, ^20^ ^]^ Therefore, we only used mouse tail skin for the observation of vitiligo progression. 34 days after vitiligo induction, exosomes derived from hUMSCs in 2D or 3D culture conditions were intravenously infused once a week respectively (Figure 3A). Initially, we confirmed the successful entry of exosomes into vitiligo mice. Subsequently, we intravenously injected 2D‐Exos and 3D‐Exos labeled with DIR and performed optical imaging. Whole‐body imaging was conducted 24 h post tail vein injection, as shown in Figure S1C (Supporting Information). The brightest signal was observed in the tail region of vitiligo mice, followed by the corresponding position of the liver. This indicates the successful injection of exosomes into vitiligo mice, with predominant accumulation observed in the tail region. A dose of 50 µg of exosomes was administered per mouse, with PBS treatment used as the control. After 10 weeks of treatment, we analyzed and quantified the depigmented lesional areas on the tails in vitiligo mice. Compared with PBS group, the areas of depigmentation in the tails of 2D‐Exos and 3D‐Exos treatment groups were much smaller, and the 3D‐Exos‐treated group was more prominent, which was almost the same as at the beginning of treatment. Meanwhile, the mean percentage area of vitiligo lesions to total tail area was also found significantly decreased in 3D‐Exos treated mice as compared to 2D‐Exos or PBS treated vitiligo mice (Figure 3C,D). In addition, whole‐mount immunofluorescence staining was used to detect the cells infiltration in mouse tail epidermis, and significant CD8^+^ T cells infiltration and melanocyte loss were observed in PBS‐treated group. Whereas, fewer CD8^+^T cells infiltration and melanocyte loss were detected in 2D‐Exos or 3D‐Exos‐treated groups (Figure 3E). Quantitative analysis confirmed that 3D‐Exos‐treated group showed an enhanced ability to reduce the ratio of CD8^+^ T lymphocytes/melanocyte in mouse tail epidermis skin (Figure 3F). Therefore, both 2D‐Exos and 3D‐Exos could reduce the extent of depigmentation in vitiligo mice by alleviating the infiltration of CD8^+^T lymphocytes in lesional site, and 3D‐Exos was more effective.

### 2.4 3D‐Exos Treatment Inhibited CD8^+^T Cells whereas Activated Treg Cells in Vitiligo Mice

**Figure 4:**
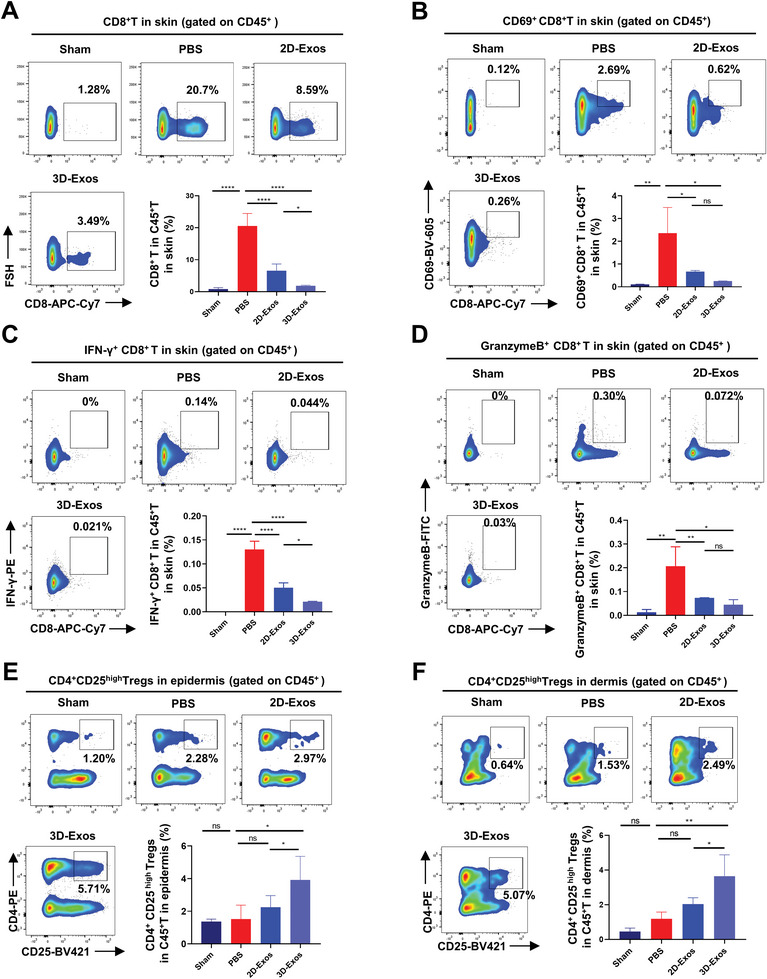
Effect of hUMSCs‐Exos on the CD8^+^T and Treg cells in the skin of vitiligo mice. A) Flow cytometry and statistical analysis of the effect of PBS, 2D‐Exos, or 3D‐Exos on CD8^+^T cells proportions in skin (*n* = 3 per group). B–D) Flow cytometry and statistical analysis demonstrated the effects of PBS, 2D‐Exos, or 3D‐Exos on the expression levels of the activation markers CD69, IFN‐γ, and GranzymeB in CD8^+^T cells in skin (*n* = 3 per group). E,F) Flow cytometry and statistical analysis revealed the impact of PBS, 2D‐Exos, or 3D‐Exos on Treg cell proportions in both epidermis and dermis (*n* = 3 or 4 per group). All sham mice were only treated with PBS (*n* = 3 or 4 per group). Error bars represented mean ± SD. Statistical significance was denoted as ^*^ *p* < 0.05, ^**^ *p* < 0.01, ^***^ *p* < 0.001, and ^****^ *p* < 0.0001 by one way analysis of variance (ANOVA) and Tukey's test; ns indicates not statistically significant.

Early vitiligo lesions are characterized by focal infiltration of CD8^+^ T lymphocytes and loss of melanocytes in skin. Besides, the number and function of regulatory T cells (Tregs) which actively suppress CD8^+^ T cells‐mediated immune responses is observed to be suppressed in vitiligo.^[^ ^21^ ^]^ In this regard, the dysregulation of both the number and function of CD8^+^ T cells and Tregs greatly accounts for the abnormal autoimmue response in vitiligo.^[^ ^22^ ^]^ Therefore, we further evaluated the effect of hUMSCs‐Exos on CD8^+^ T and Treg cells infiltration and function in vitiligo mice by using flow cytometry. As was shown, significant reduction in the number of infiltrated CD8^+^T cells in the skin and blood of both 2D‐Exos and 3D‐Exos‐treated vitiligo mice was observed compared to PBS control. More importantly, 3D‐Exos exhibited a superior effect in reducing the number of infiltrated CD8^+^T cells compared to 2D‐Exos group (**Figure** **4**A; Figure S2A, Supporting Information), which is consistent with the results of CLSM shown above. Additionally, 3D‐Exos markedly attenuated the expression levels of the activation marker cluster of differentiation 69 (CD69), interferon‐gamma (IFN‐γ), and Granzyme B in CD8^+^ T cells in both the tail skin and peripheral blood of vitiligo mice (Figure 4B–D; Figure S2B–D, Supporting Information). Moreover, we found that recruitment of CD4^+^CD25^high^ Tregs was significantly increased by nearly two‐fold in the skin of 3D‐Exos‐treated mouse compared with PBS group, while the 2D‐Exos‐treated group only showed a slightly increased proportion of CD4^+^CD25^high^ Tregs (Figure 4E,F). Meanwhile, we observed a significant increase in the number of CD4^+^Foxp3^+^ Tregs in the peripheral blood of vitiligo mice treated with 2D‐Exos and 3D‐Exos compared to the PBS group, higher in 3D‐Exos treatment group (Figure S2E, Supporting Information). Furthermore, we assessed the expression of relevant suppressive effector molecules in Tregs, revealing that 3D‐Exos significantly promoted the expression of interleukin‐10 (IL‐10) and transforming growth factor beta (TGF‐β) in Tregs compared to both the PBS and 2D‐Exos groups (Figure S2F,G, Supporting Information). Moreover, we also detected the effect of hUMSCs‐Exos on the proportion of CD8^+^T cells and Tregs in spleen, which displayed no prominent alteration among the PBS, 2D‐Exos, and 3D‐Exos treatment groups (Figure S2H,I, Supporting Information). Therefore, while excessive CD8^+^T cells infiltration and reduced Tregs proportion contribute to the progression of vitiligo in mice, treatment with 3D‐Exos resulted in reduced infiltration and activation of CD8^+^T cells, and simultaneously expanded the Tregs population and potentiated their immune‐suppressive function in vitiligo mice.

### 2.5 3D‐Exos Treatment Expanded the Population of Treg Cells and Restored their Function In *Vitro*

**Figure 5:**
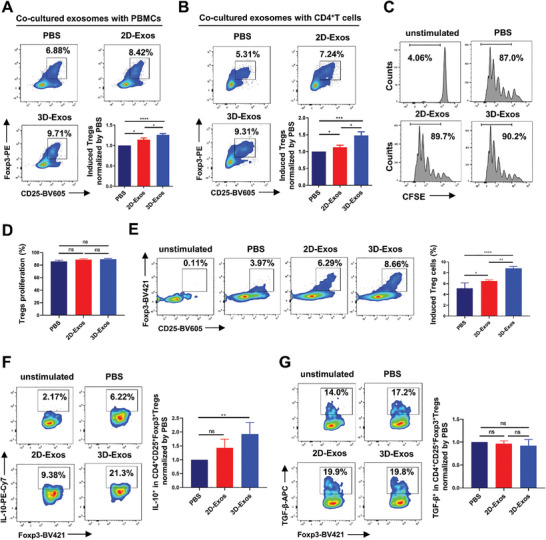
3D‐Exos treatment led to an expansion of Treg cell population and reinstated their function in vitro. A) Comparison of PBS, 2D‐Exos, and 3D‐Exos effects on Treg cell proportion in PBMCs with quantification, all groups normalized to PBS group (*n* = 6 per group). B) The impact of PBS, 2D‐Exos, and 3D‐Exos on Treg cell proportion in CD4^+^T cells with quantification, all groups normalized to PBS group (*n* = 6 per group). C,D) CFSE‐labeled Tregs were cultured with or without 2D‐Exos and 3D‐Exos for 5 days in the presence of CD3/CD28 microbeads. Proliferating cell percentage was evaluated via flow cytometry (*n* = 3 per group). E) naïve CD4^+^T cells were co‐cultured with or without 2D‐Exos and 3D‐Exos in medium containing IL‐2 and TGF‐β. Flow cytometry was performed to detect the proportion of Treg cells after 5 days of stimulation with CD3/CD28 beads (*n* = 4 per group). F,G) PBMCs were cultured with or without 2D‐Exos and 3D‐Exos for 48 hours in the presence of CD3/CD28 microbeads. The percentage of IL‐10 and TGF‐β expression in Treg cells was evaluated by flow cytometry, with all groups normalized to the PBS group (*n* = 4 per group). Error bars represented mean ± SD. Statistical significance was denoted as ^*^ *p* < 0.05, ^**^ *p* < 0.001, ^***^ *p* < 0.001, and ^****^ *p* < 0.0001 by one‐way analysis of variance (ANOVA) and Tukey's test; ns indicates not statistically significant.

Tregs helps to keep the immune response in balance by suppressing the activation of autoreactive CD8^+^T cells.^[^ ^1^ ^]^ It is found that increasing the number of Tregs result in a continuous remission of depigmentation in mice with vitiligo,^[^ ^23^ ^]^ indicating that replenishing Tregs can repair the disrupted autoimmune tolerance and is a promising therapeutic method for vitiligo. We then asked whether hUMSCs‐derived exosomes could induce the activation and proliferation of Tregs in vitro. Initially, peripheral blood mononuclear cells (PBMCs) were obtained from the healthy donors. To verify whether hUMSCs‐derived exosomes can be internalized by PBMCs, we co‐cultured PKH26‐labeled 3D‐Exos with PBMCs for 24 h and observed the results using CLSM, which showed that the exosomes were successfully internalized by PBMCs (Figure S3A, Supporting Information). Then, 2D‐Exos, 3D‐Exos, or PBS were incubated with PBMCs for 3 days, and Tregs were subsequently analyzed by flow cytometry. Interestingly, in the absence of cytokine‐based induction media, compared with the PBS group, hUMSCs‐Exos significantly increased the number and percentage of Tregs, and the 3D‐Exos‐treated group offered a better effect on inducing the expansion of Tregs than that of 2D‐Exos‐treated group (**Figure** **5**A). Next, to ensure the effect was due to the induction from CD4^+^T cells instead of affecting other immune cells, we therefore repeated the experiment by fostering CD4^+^T cells isolated from PBMCs together with 2D‐Exos, 3D‐Exos, or PBS. Consistently, compared to the PBS and the 2D‐Exos, 3D‐Exos markedly increased Tregs population in CD4^+^T cells (Figure 5B). These results suggested that 3D‐Exos treatment could induce Tregs expansion. Thereafter, we went on to investigate the mechanism underlying the expansion of Tregs. First, Treg cells isolated from PBMCs of healthy individuals were confirmed to have a purity of 92.8% using flow cytometry (Figure S3B, Supporting Information). Subsequently, the isolated Treg cells were co‐cultured with 2D‐Exos and 3D‐Exos, during which CD3/CD28 activation magnetic beads were utilized to stimulate Treg cell proliferation.^[^ ^24^ ^]^ The results indicated that, compared to the PBS group, both 2D‐Exos and 3D‐Exos had no effect on Treg cell proliferation (Figure 5C,D). Following this, to assess whether hUMSCs‐Exos influence T cell differentiation, we isolated naïve CD4^+^T cells from healthy individuals' PBMCs. Subsequently, naïve CD4^+^T cells were co‐cultured with 2D‐Exos and 3D‐Exos in RPMI 1640 medium supplemented with interleukin‐2 (IL‐2) and TGF‐β. After stimulation with CD3/CD28 activation magnetic beads for 5 days, changes in the proportion of Treg cells were assessed. Results indicated that the proportion of naïve CD4^+^T cells differentiating into Treg cells in the absence of CD3/CD28 activation magnetic beads was minimal (0.11%). However, compared to the PBS group, both the 2D‐Exos and 3D‐Exos groups facilitated Treg cell differentiation, with the 3D‐Exos group exhibiting superior promotion (Figure 5E). This suggests that 3D‐Exos more effectively promoted the differentiation of naïve CD4^+^T cells into Treg cells. Next, we employed flow cytometry to assess the impact of hUMSCs‐Exos on the expression of relevant suppressive effector molecules in Treg cells, including IL‐10 and TGF‐β. The results indicate that 3D‐Exos significantly enhanced the proportion of IL‐10^+^Treg cells (Figure 5F), while showing no significant effect on the proportion of TGF‐β^+^Treg cells (Figure 5G). The above results indicate that 3D‐Exos could enhance immune suppressive ability by more efficiently promoting Treg cell differentiation and the expressions of immunosuppressive effector molecule. Meanwhile, we also conducted relevant experiments in PBMCs from vitiligo patients, which showed that 3D‐Exos had no significant impact on Treg cell proliferation (Figure S4A, Supporting Information). However, 3D‐Exos promoted the differentiation of naïve CD4^+^T cells into Tregs (Figure S4B, Supporting Information), and significantly enhanced the proportion of IL‐10^+^Treg cells and TGF‐β^+^Treg cells (Figure S4C,D, Supporting Information). Next, we verified whether 3D‐Exos could restore the inhibitory function of Treg cells in CD8^+^T cells in vitiligo. First, Tregs and CD8^+^T cells were isolated from PBMCs in vitiligo patients, subsequently co‐cultured with or without 3D‐Exos, and then the proliferation and activation of CD8^+^T cells were detected (Figure S4E, Supporting Information). The results showed that compared to adding Treg cells alone, the 3D‐Exos group significantly enhanced the ability of Treg cells to inhibit CD8^+^T cell proliferation (Figure S4F, Supporting Information). At the same time, 3D‐Exos also enhanced the ability of Treg cells to inhibit surface activation molecule CD69 on CD8^+^T cells (Figure S4G, Supporting Information). What is more, we used Tregs pre‐treated with 3D‐Exos or not to co‐culture with CD8^+^T cells, which revealed that 3D‐Exos‐stimulated Tregs could more effectively suppress the activation of CD8^+^T cells (Figure S4H, Supporting Information), and indicated that Treg was attributed to the suppressive effect of 3D‐Exos on CD8^+^T cells (Figure S4I, Supporting Information).

### 2.6 3D‐Exos Alleviated Oxidative Stress and Inhibited Apoptosis in H_2_O_2_‐Stimulated Vitiligo Melanocytes

**Figure 6:**
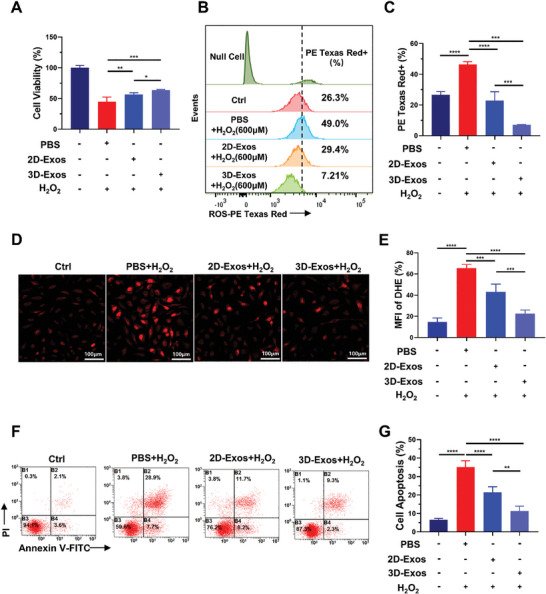
hUMSCs‐Exos pre‐treatment reduced oxidative stress and apoptosis in H_2_O_2_‐treated PIG3V cells. A) CCK‐8 assay was used to detect the effect of pretreatment with hUMSCs‐Exos on the cell viability of PIG3V cells treated with H_2_O_2_ (*n* = 3). B–E) Flow cytometry analysis (B,C) and immunofluorescence detection (D,E) of the intracellular ROS using fluorescent probe DHE (PE‐Texas‐Red) (*n* = 3). F,G) Apoptotic (annexin V+/PI−) and necrotic (annexin V+/PI+) PIG3V cells were identified by flow cytometry (*n* = 4). Error bars represented mean ± SD. Statistical significance was denoted as ^*^ *p* < 0.05, ^**^ *p* < 0.01, ^***^ *p* < 0.001, and ^****^ *p* < 0.0001 by one‐way analysis of variance (ANOVA) and Tukey's test (A,C,E,G).

We next sought to evaluate whether hUMSCs‐Exos could directly affect the viability of human melanocytes. Different concentrations of 2D‐Exos and 3D‐Exos (5, 10, and 20 µg mL^−1^) were added to co‐incubate with PIG3V cells for 48 h, and cell viability of PIG3V cells was evaluated by Cell Counting Kit‐8 (CCK‐8) assay. As was shown, hUMSCs‐Exos treatment resulted in increased melanocytes viability at the concentration of 10 or 20 µg mL^−1^, and 3D‐Exos significantly promoted cells proliferation compared to 2D‐Exos or control group (Figure S5A, Supporting Information). Since no significant difference was observed in cell viability after administration of 3D‐Exos over 10 µg mL^−1^, exosomes at the concentration of 10 µg mL^−1^ were finally selected for subsequent experiments. Notably, our preceding research has demonstrated that oxidative stress can efficiently activate local skin innate immune response and then initiate melanocyte‐specific T‐cell immune response in vitiligo.^[^ ^2^, ^25^ ^]^ Moreover, it can also directly induce the cell death of melanocytes.^[^ ^26^ ^]^ Therefore, we then explored the anti‐oxidative and pro‐survival effect of hUMSCs‐Exos on melanocytes under H_2_O_2_‐induced oxidative stress. To simulate oxidative stress in vitro, we investigated the effects of different concentrations of H_2_O_2_ on PIG3V cells viability by using CCK8 assay, which showed that treatment with H_2_O_2_ at a concentration above 600 µm for 24 h significantly inhibited PIG3V cells viability, and thus 600 µm H_2_O_2_ was selected for the following experiments (Figure S5B, Supporting Information). As was shown, pretreatment with 2D‐Exos or 3D‐Exos could protect vitiligo melanocytes from H_2_O_2_‐mediated toxicity, and 3D‐Exos exhibited a much better effect than 2D‐Exos (**Figure** **6**A). To determine whether hUMSCs‐Exos modulated the level of reactive oxygen species (ROS) generated in vitiligo melanocytes in response to H_2_O_2_ treatment, we measured the intracellular level of ROS by using fluorescent probe DHE (PE‐Texas‐Red). As shown in Figure 6B,D, treatment with H_2_O_2_ induced a robust increase in DHE fluorescence level, whereas pre‐treatment with 2D‐Exos or 3D‐Exos for 48 h dramatically decreased the fluorescence intensity. Quantitative analysis confirmed that hUMSCs‐Exos treatment significantly reduced the H_2_O_2_‐induced ROS accumulation in vitiligo melanocytes, and 3D‐Exos had a greater ROS scavenging ability (Figure 6C,E). To further figure out whether the reduction of cell viability was due to the induction of apoptosis, we performed flow cytometry analysis to assess the apoptosis rates of vitiligo melanocytes treated with 2D‐Exos or 3D‐Exos before the cells were exposed to H_2_O_2_. Compared with the PBS and 2D‐Exos‐treated groups, the proportion of apoptotic cells was significantly reduced upon 3D‐Exos treatment (Figure 6F,G). These data suggest that hUMSCs‐Exos exerted anti‐oxidative and anti‐apoptotic functions in response to oxidative stress. Importantly, these cyto‐protective effects were superior for 3D‐Exos compared with 2D‐Exos.

### 2.7 MiR‐132‐3p and miR‐125b‐5p were Key Mediators of the Immune‐Modulatory and Anti‐Oxidative Capability of 3D‐Exos

**Figure 7:**
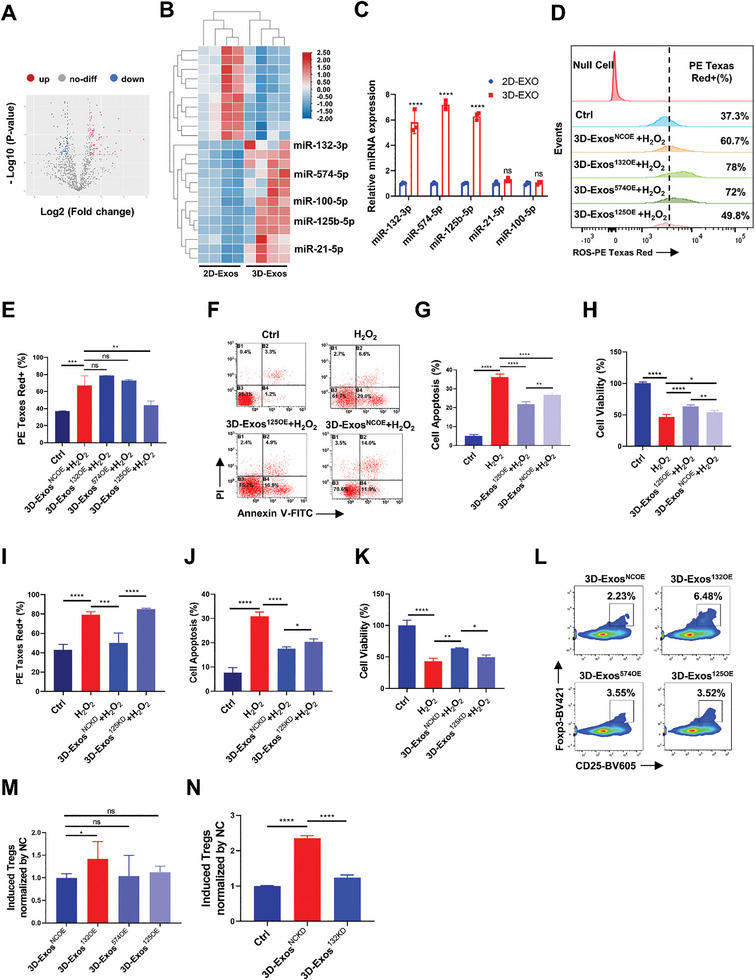
3D‐Exos exerted anti‐oxidative and immunosuppressive effects by delivering miR‐125b‐5p and miR‐132‐3p. A) Volcano plots illustrated the differentially expressed miRNAs between 2D‐Exos and 3D‐Exos. B) Heatmap of differentially expressed miRNAs between 2D‐Exos and 3D‐Exos. C) RT‐PCR analysis of the levels of candidate miRNAs in 2D‐Exos and 3D‐Exos (*n* = 3 per group). D,E) The impact of overexpressing different miRNAs in 3D‐Exos on intracellular ROS levels in PIG3V cells was analyzed by flow cytometry (*n* = 3 per group). F,G) Effects of 3D‐Exos^NCOE^ or 3D‐Exos^125OE^ on cell apoptosis of PIG3V cells were evaluated through flow cytometry analysis (*n* = 3 per group). H) Effects of 3D‐Exos^NCOE^ or 3D‐Exos^125OE^ on cell viability under H_2_O_2_ treatment were assessed using the CCK8 assay (*n* = 4 per group). I–K) Statistical analysis was conducted to assess the effects of 3D‐Exos^NCKD^ or 3D‐Exos^125KD^ on intracellular ROS levels, cell apoptosis, and cell viability under H_2_O_2_ treatment (*n* = 3 or 4 per group) in PIG3V cells. L,M) The impact of overexpressing different miRNAs in 3D‐Exos on proportion of Treg cells in CD4^+^T cells (*n* = 3 per group). N) Statistical analysis of the effects of 3D‐Exos^NCKD^ or 3D‐Exos^132KD^ on proportion of Treg cells in CD4^+^T cells (*n* = 3 per group). Error bars represented mean ± SD. Statistical significance was denoted as ^*^ *p* < 0.05; ^**^ *p* < 0.01; ^***^ *p* < 0.001, ^****^ *p* < 0.0001 by *t* test (C) or by one‐way analysis of variance (ANOVA) and Tukey's test (E,G–K,M,N).

As a crucial component of exosome cargo, miRNAs have been reported to be deeply involved in regulating intercellular communication and multiple biological functions. Based on the results above, we performed high‐throughput miRNA sequencing of 2D‐Exos and 3D‐Exos to identify the potential miRNAs that mediate the anti‐oxidative and immune‐modulatory effects. Compared with the 2D‐Exos group, 57 up‐regulated miRNAs and 36 down‐regulated ones were identified in the 3D‐Exos group (≥ 2‐fold, *P* < 0.05) (**Figure** **7**A). We eliminated miRNAs with relatively low abundance and selected 23 miRNAs that were differentially‐expressed in 2D‐Exos and 3D‐Exos (Figure 7B). Then, the top five miRNAs (miR‐132‐3p, miR‐574‐5p, miR‐125b‐5p, miR‐21‐5p and miR‐100‐5p) with highest fold change or expression in 3D‐Exos were selected (**Table** **1**), and three miRNAs including miR‐132‐3p, miR‐574‐5p, and miR‐125b‐5p were validated significantly up‐regulated in 3D‐Exos compared with 2D‐Exos by RT‐qPCR (Figure 7C). We forwardly went to verify the anti‐oxidative and immune‐modulatory function of these 3 miRNAs. We employed a lentivirus‐based approach to establish miR‐125b‐5p, miR‐132‐3p, and miR‐574‐5p overexpression (miR^OE^), as well as knockdown of miR‐125b‐5p and miR‐132‐3p (miR^KD^), along with corresponding negative controls (miR‐NC^OE^ and miR‐NC^KD^), in hUMSCs. Exosomes were isolated from the conditioned media of overexpressing and knockdown hUMSCs, designated as 3D‐Exos^NCOE^, 3D‐Exos^125OE^, 3D‐Exos^132OE^, 3D‐Exos^574OE^, 3D‐Exos^NCKD^, 3D‐Exos^125KD^, and 3D‐Exos^132KD^, respectively. Quantitative reverse transcription polymerase chain reaction (qRT‐PCR) results confirmed the successful establishment of hUMSCs with overexpression and knockdown of the target miRNAs (Figure S6A–E, Supporting Information). Subsequently, exosomes were isolated from their conditioned medium, and the qRT‐PCR results also confirmed the successful overexpression and knockdown of miRNAs in the exosomes, which were then utilized for subsequent experiments (Figure S6F–J, Supporting Information). Specifically, only 3D‐Exos^125OE^ significantly reduced ROS levels in H_2_O_2_‐stimulated vitiligo melanocyte, whereas the other two stimulations exerted no prominent alteration (Figure 7D,E). In consistent, 3D‐Exos^125OE^ reversed H_2_O_2_‐induced cell viability suppression and apoptosis (Figure 7F–H). Subsequently, loss‐of‐function studies revealed that downregulation of miR‐125b‐5p levels significantly attenuated the protective effect of 3D‐Exos, characterized by decreased cell viability, elevated ROS levels, and increased apoptosis (Figure 7I‐7K; Figure S6K,L, Supporting Information). Therefore, miR‐125b‐5p was responsible for the anti‐oxidative and pro‐survival effects of 3D‐Exos on melanocytes. Furthermore, to co‐culture 3D‐Exos overexpressing miR‐132‐3p, miR‐574‐5p, and miR‐125b‐5p individually with CD4^+^T cells for 3 days, followed by stimulation with CD3/CD28 activation magnetic beads for 24 hours, resulted in a significant increase in the percentage of Tregs in CD4^+^T cells in 3D‐Exos^132OE^ group compared with the 3D‐Exos^NCOE^ group (Figure 7L,M). Functional loss studies demonstrated that knockdown of miR‐132‐3p in 3D‐Exos significantly abrogated the ability of 3D‐Exos to promote Tregs expansion (Figure 7N; Figure S6M, Supporting Information). Therefore, miR‐132‐3p was responsible for the immune‐modulatory effect of 3D‐Exos on Tregs. Overall, these findings demonstrates that miR‐125b‐5p and miR‐132‐3p were key mediators of the anti‐oxidative and immune‐modulatory capability of 3D‐Exos in vitiligo treatment.

**Table 1:** Differentially‐expressed miRNAs in 2D‐Exos and 3D‐Exos.

MiRNA ID	2D‐Exos	3D‐Exos	Fold change	*P* value	Up/Down
hsa‐miR‐132‐3p	1514	5678	3.75	0.040657	up
hsa‐miR‐574‐5p	2192	5616	2.56	0.0314241	up
hsa‐miR‐125b‐5p	61 229	142 823	2.33	0.011946	up
hsa‐miR‐199b‐5p	4449	10 137	2.28	0.049639	up
hsa‐miR‐196a‐5p	1864	4163	2.23	0.025865	up
hsa‐miR‐503‐5p	5019	11 157	2.22	0.002213	up
hsa‐miR‐193a‐5p	8668	19 123	2.21	0.044640	up
hsa‐miR‐92b‐3p	2096	4562	2.18	0.001628	up
hsa‐miR‐100‐5p	81947	176869	2.16	0.008639	up
hsa‐miR‐181b‐5p	3331	7031	2.11	0.006248	up
hsa‐miR‐21‐5p	1293933	2 723 351	2.10	0.033695	up
hsa‐miR‐432‐5p	3711	7749	2.09	0.043811	up
hsa‐miR‐125a‐5p	4525	9123	2.02	0.028602	up

^a)^ 2D‐Exos: exosomes isolated from hUMSCs cultured under 2D conditions;

^b)^ 3D‐Exos: exosomes isolated from hUMSCs cultured under 3D conditions.

### 2.8 *Bak1* and *Sirt1* are Potential Targets of miR‐125b‐5p and miR‐132‐3p Respectively

**Figure 8:**
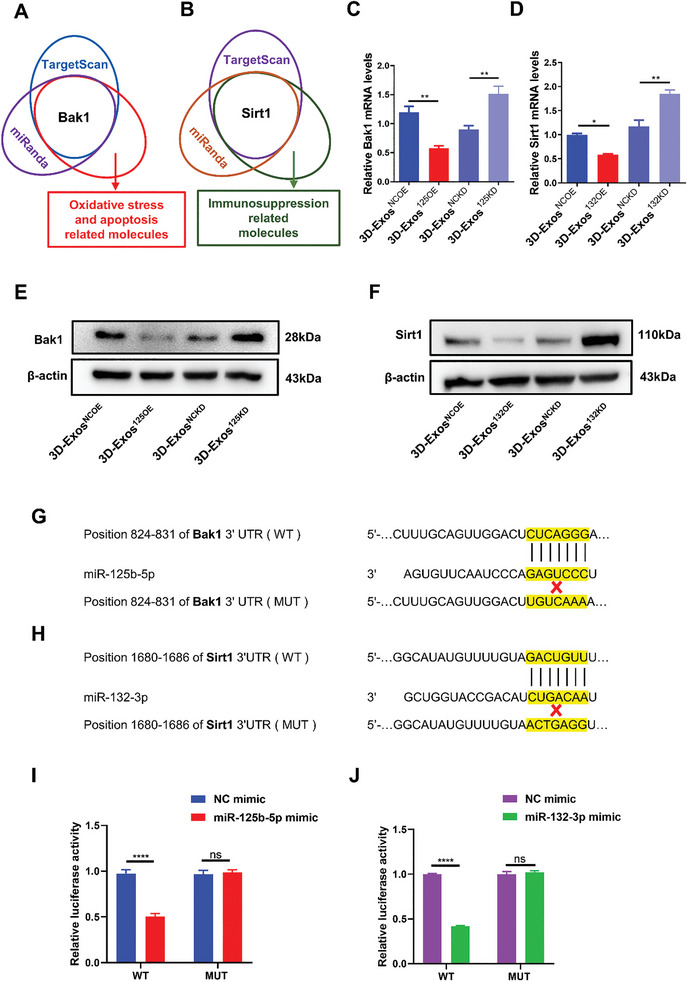
*Bak1* and *Sirt1* were direct target genes of miR‐125b‐5p and miR‐132‐3p, respectively. A,B) Screening scheme for putative target genes that might contribute to the anti‐oxidative and anti‐apoptotic effects of miR‐125b‐5p, and the immunosuppressive effects of miR‐132‐3p. C,E) The mRNA and protein levels of *Bak1* in PIG3V cells after the transfection with 3D‐Exos^NCOE^, 3D‐Exos^125OE^, 3D‐Exos^NCKD^, 3D‐Exos^125KD^ for 48 h (*n* = 3 per group). D,F) The mRNA and protein levels of *Sirt1* in CD4^+^T cells after the transfection with 3D‐Exos^NCOE^, 3D‐Exos^132OE^, 3D‐Exos^NCKD^, 3D‐Exos^132KD^ for 48 h (*n* = 3 per group). G,H) Targetscan revealed that miR‐125‐5p and miR‐132‐3p could bind to the target genes *Bak1* and *Sirt1*, respectively. I,J) Luciferase reporter assay presented the luciferase activity of wild‐type (WT) and mutant (MUT) *Bak1* or *Sirt1* in 293T cells, after the transfection with miR‐125b‐5p mimic and miR‐132‐3p mimic respectively (*n* = 3 per group). Error bars represented mean ± SD. Statistical significance was denoted as ^*^ *p* < 0.05, ^**^ *p* < 0.01, and ^****^ *p* < 0.0001 by one‐way analysis of variance (ANOVA) and Tukey's test (C,D) or by t test (I,J); ns indicates not statistically significant.

Target genes of hsa‐miR‐125b‐5p and hsa‐miR‐132‐3p predicted by miRanda and TargetScan were further analyzed by the Gene Ontology (GO) database (http://geneontology.org) and Kyoto Encyclopedia of Genes and Genomes pathway (KEGG) pathway database (http://www.genome.jp/kegg). It can be seen from the GO and KEGG pathways enrichment scatter plot that the target genes of miR‐125b‐5p are enriched in oxidative stress and apoptosis‐related pathways (Figure S8A,B, Supporting Information), while the target genes of miR‐132‐3p are enriched in T cell immune response related pathways (Figure S8C,D, Supporting Information). Among the possible miR‐125b‐5p target genes, we focused on *Bak1*, which is involved in oxidative stress injury and apoptotic pathways (**Figure** **8**A). Meanwhile, *Sirt1* that is involved in driving the abundance of Treg cells by directly regulating the acetylation level of Foxp3 protein and increasing Foxp3 mRNA production was selected as the possible target gene of miR‐132‐3p(Figure 8B).^[^ ^27^ ^]^ Furthermore, we found that 3D‐Exos^125OE^ significantly decreased *Bak1* mRNA and protein levels, while the 3D‐Exos^125KD^ increased *Bak1* mRNA and protein levels in PIG3V cells (Figure 8C,E). Similar results showed that 3D‐Exos^132OE^ significantly reduced *Sirt1* expression, while 3D‐Exos^132KD^ increased *Sirt1* mRNA and protein levels in CD4^+^T cells (Figure 8D,F). Then, the binding sites for miR‐125b‐5p in the 3′‐untranslated regions (3′UTRs) of *Bak1* and miR‐132‐3p in the 3′UTRs of *Sirt1* were further examined using a dual luciferase reporter assay. We cloned either their wild‐type 3′UTRs or mutant 3′UTRs in putative miR‐125b‐5p and miR‐132‐3p binding sites into a reporter plasmid and assessed their responsiveness to miR‐125b‐5p or miR‐132‐3p in 293T cells (Figure 8G,H). As was depicted by the data, miR‐125b‐5p and miR‐132‐3p reduced luciferase activity of *Bak1* or *Sirt1* wild‐type 3′UTR constructs, but had no effect when the *Bak1* or *Sirt1* binding sites were mutated (Figure 8I,J). Taken together, these results further confirmed that *Bak1* was the downstream target gene of miR‐125b‐5p in human melanocytes, and *Sirt1* was the downstream target gene of miR‐132‐3p in CD4^+^T cells.

### 2.9 MiR‐132‐3p Mediated the Effect of 3D‐Exos on Vitiligo In Vivo

**Figure 9:**
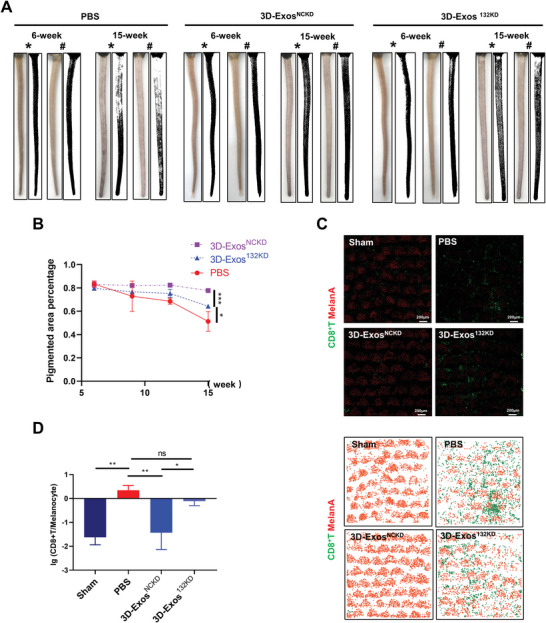
Establishment of vitiligo mouse model and tail vein administration of 3D‐Exos^132KD^. A) Representative tail images and ImageJ analysis of mice in PBS, 3D‐Exos^NCKD^, or 3D‐Exos^132KD^ group (n = 3 per group) at weeks 6 and 15 (* dorsal, # ventral). B) The tail pigmentation percentages of mice in the three groups in 10 consecutive weeks (from week 6 to week 15) was calculated using Image J software (*n* = 3 per group). C) Whole‐mount immunofluorescent staining images of CD8^+^T cells (green) and melanocytes (red), and corresponding heatmaps of CD8^+^T cells (green) and melanocytes (red) in the tail epidermis of vitiligo mice (*n* = 3 per group). D) Statistical analysis of lg value of the number of CD8^+^T/melanocyte in whole‐mount immunofluorescent staining. All sham mice were only treated with PBS. Error bars represented mean ± SD. Statistical significance was denoted as ^*^ *p* < 0.05, ^**^ *p* < 0.01, and ^***^ *p* < 0.001 by one‐way analysis of variance (ANOVA) and Tukey's test (B,D).

**Figure 10:**
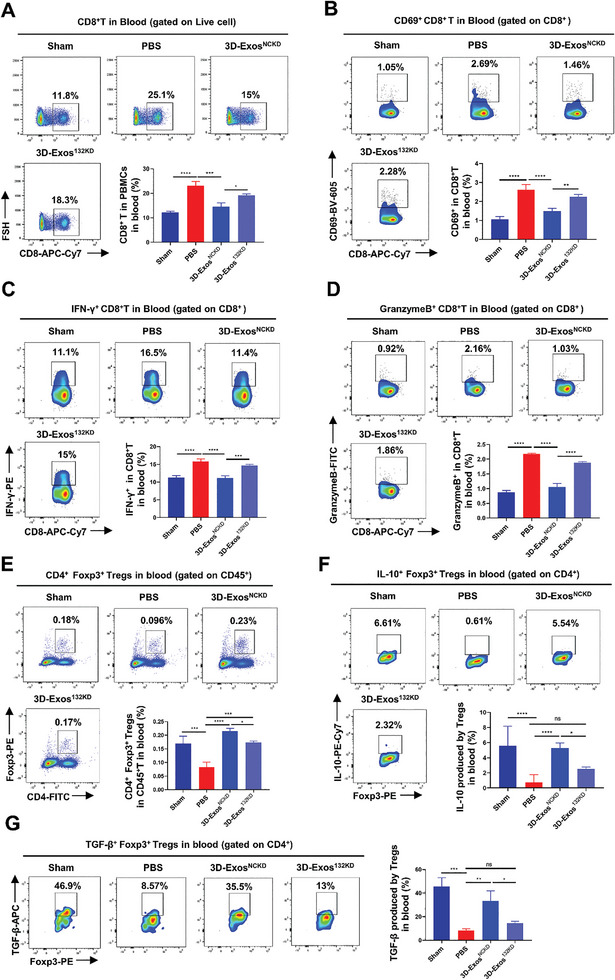
Effect of 3D‐Exos^132KD^ on the CD8^+^T and Treg cells in the blood of vitiligo mice. A) Flow cytometry and statistical analysis of the effect of PBS, 3D‐Exos^NCKD^, or 3D‐Exos^132KD^ on CD8^+^T cells proportions in blood (*n* = 3 per group). B–D) Flow cytometry and statistical analysis were performed to assess the effects of PBS, 3D‐Exos^NCKD^, or 3D‐Exos^132KD^ on the expression levels of the activation markers CD69, IFN‐γ, and GranzymeB in CD8^+^T cells in blood (*n* = 3 or 4 per group). E) Flow cytometry and statistical analysis of the effect of PBS, 3D‐Exos^NCKD^, or 3D‐Exos^132KD^ on Treg cells proportions in blood (*n* = 3 or 4 per group). F,G) Flow cytometry and statistical analysis of the effect of PBS, 3D‐Exos^NCKD^, or 3D‐Exos^132KD^ on the expression of IL‐10 and TGF‐β in Treg cells in blood (*n* = 3 per group). All sham mice were only treated with PBS (*n* = 3 or 4 per group). Error bars represented mean ± SD. Statistical significance was denoted as ^*^ *p* < 0.05, ^**^ *p* < 0.01, ^***^ *p* < 0.001, and ^****^ *p* < 0.0001 by one‐way analysis of variance (ANOVA) and Tukey's test; ns indicates not statistically significant.

Thereafter, we went on to investigate whether miR‐132‐3p mediated the effect of 3D‐Exos on vitiligo in vivo. To this end, after 34 days of vitiligo induction, mice in each group were intravenously injected with either 3D‐Exos^NCKD^ or 3D‐Exos^132KD^, with PBS as a control. After the treatment duration of 10 weeks, the depigmented area in the tail of vitiligo mice was analyzed. Compared to the PBS group, the average percentage of depigmented area in the tail of mice treated with 3D‐Exos^NCKD^ was significantly reduced, whereas mice injected with 3D‐Exos^132KD^ exhibited a notable increase in depigmented area in the tail (**Figure** **9**A,B). Immunofluorescence staining revealed significant infiltration of CD8^+^T cells and a marked reduction in melanocytes in tail epidermis of mice treated with PBS. However, fewer CD8^+^T cell infiltrations and less melanocytes loss were observed in the 3D‐Exos^NCKD^‐treated group, which was largely reversed in the 3D‐Exos^132KD^ group (Figure 9C,D). Hence, these data demonstrates that depletion of miR‐132‐3p in 3D‐Exos attenuated the therapeutic effect of 3D‐Exos on depigmentation in vitiligo mice. We further examined the changes in the proportion and function of CD8^+^T cells and Treg cells in the skin and peripheral blood of vitiligo mice treated with 3D‐Exos with or without the depletion of miR‐132‐3p. As was shown, treatment with 3D‐Exos^NCKD^ significantly reduced the proportion of CD8^+^T cells in the skin and peripheral blood of mice, and inhibited the expression of the activation marker CD69, IFN‐γ and Granzyme B (**Figure** **10**A–D; Figure S7A–D, Supporting Information). However, the inhibitory effect was significantly ameliorated in the 3D‐Exos^132KD^ group. Additionally, compared to PBS, treatment with 3D‐Exos^NCKD^ could increase the proportion and suppressive function of Treg cells in the peripheral blood of vitiligo mice (Figure 10E,G), whereas the effect was also abrogated in 3D‐Exos^132KD^ group. The above results demonstrated that miR‐132‐3p mediates the effect of 3D‐Exos on vitiligo in vivo via the regulation of Tregs infiltration and function.

## 3 Discussion

Over the past few decades, an increasing number of clinical studies^[^ ^28^ ^]^ have demonstrated that hUMSCs‐based therapy is a novel potent strategy for the treatment of various autoimmune diseases due to their beneficial functions including the regulation of abnormal immune response, the inhibition of cell apoptosis, and the scavenge of ROS,^[^ ^4^, ^5^ ^]^ whereas some disadvantages like immune rejection, undirected cell differentiation and tumorigenicity significantly limit their wide clinical applications.^[^ ^7^, ^29^ ^]^ In contrast to hUMSCs, hUMSCs‐Exos are important bioactive EVs with relatively low immunogenicity, while still harbor comparable biological activities, as well as of high stability and a rich source,^[^ ^30^ ^]^ making them promising cell‐free therapeutic choice which has attracted considerable attention for the treatment of various autoimmune skin diseases like vitiligo.

However, exosome‐based therapy has not yet been translated to clinical practice due to several major obstacles such as low production yield and limited cytoprotective ability. Thus, optimizing hUMSCs‐Exos in vitro to enhance their therapeutic effects is of great importance. Therefore, several strategies have been previously developed to improve the therapeutic potentials, such as gene modification,^[^ ^31^ ^]^ hypoxia pre‐conditioning,^[^ ^32^ ^]^ and pharmacological pre‐treatment.^[^ ^33^ ^]^ However, all the above methods are conducted in a traditional 2D monolayer culture condition that makes hUMSCs endure limited expansion, phenotypic changes, and loss of cellular therapeutic activity during long‐term passaging.^[^ ^29^ ^]^ Intriguingly, 3D culture of hUMSCs enables mass production and is advantageous to improve their paracrine function. Several 3D‐ culture systems have been developed and spheroid clusters of MSCs formed by self‐assembly represent one of the best models for the 3D culture.^[^ ^34^ ^]^ MSCs within the spheroid are naturally exposed to hypoxia, which preconditions an ischemic environment, and exhibit more EVs section and higher therapeutic potential.^[^ ^34^, ^35^ ^]^ Here, we used ultralow attachment plates to establish 3D spheroids culture system, and found that in the process of multi‐hUMSCs accumulation, 3D‐hUMSCs spheres were gradually formed that could help keep the phenotype of hUMSCs and maintain their stemness.

The functional compositions of MSCs‐Exos are also highly influenced by different physiological conditions, suggesting that changes in the microenvironment in which their original cells reside could induce a modification of the exosomal paracrine and content.^[^ ^17^ ^]^ Previous studies have shown that 3D‐cultured MSCs‐derived exosomes yield more than 2D‐cultured MSCs‐derived exosomes.^[^ ^16b–d^ ^]^ In the present study, we collected 2D‐hUMSCs and 3D‐hUMSCs culture supernatants in corresponding to same numbers of cells and then obtained exosomes, of which the particle size, morphology and surface marker expression were nearly identical, whereas the quantity of exosomal protein from 3D‐hUMSCs culture supernatant was significantly higher than that from 2D‐hUMSCs, indicating that 3D culture could potentiate the exosome paracrine function of hUMSCs. These results are consistent with previous reports that high yield of exosomes can be achieved from hUMSCs under 3D culture. As hypoxia has been reported to promote exosome secretion in monolayer culture,^[^ ^36^ ^]^ the development of hypoxic environment in spheroids appears to be one of the underlying mechanisms responsible for the increased exosome production under 3D culture condition.^[^ ^37^ ^]^ Moreover, differences in cellular uptake between 3D‐Exos and 2D‐Exos may be explained by different contents of some specific proteins. Research suggests that Fetuin A and ICAM‐1 are both crucial protein molecules mediating the adhesion between exosome and cells. Exosomes produced and released by tumor cells without Fetuin A fail to promote the swift dissemination of cells.^[^ ^38^ ^]^ When ICAM‐1 on Tumor‐derived extracellular vesicles (TEVs) is obstructed, their engagement with CD8^+^T cells diminishes, thereby alleviating the inhibitory effects mediated by PD‐L1 on TEVs.^[^ ^39^ ^]^ Consistent with our findings, compared to 2D‐Exos, Fetuin A and ICAM‐1 expression is upregulated in 3D‐Exos, which may account for the enhanced uptake of 3D‐Exos by PIG3V cells. The in vivo and in vitro experiments in the present study evidently demonstrated that exosomes derived from hUMSCs cells under 3D culture condition showed better biological functions than that from 2D culture condition, which was in line with previous reports,^[^ ^14^, ^17^ ^]^ highlighting the notion that exosomes produced from 3D culture are more biologically active. In particular, a vitiligo mouse model was established and gross evaluation of mouse tail depigmentation revealed that the 3D‐Exos treatment group displayed more prominent suppression of the onset and progression of depigmentation compared to 2D‐Exos group. Previous studies have shown that the infiltration of CD8^+^ T cells accounts for a cardinal mechanism responsible for melanocyte immune destruction in vitiligo.^[^ ^3^, ^40^ ^]^ In consistent, the enhanced infiltration and function of Tregs that can help terminate abnormal activation of CD8^+^ T cells also plays a critical role, which is supported by the evidence that the increased number of Treg cells was inversely proportional to the severity of vitiligo,^[^ ^23^ ^]^ the number of Treg cells in peripheral blood of patients with active vitiligo was lower than patients with stable vitiligo,^[^ ^21^ ^]^ and more importantly, either adoptive transfer of Treg cells or pharmacology‐induced increased abundance of Treg cells significantly reduced depigmentation in h3TA2 vitiligo mice.^[^ ^41^ ^]^ Therefore, to replenish Treg cells is a promising therapeutic approach for vitiligo by restraining dysregulated autoimmunity and suppressing depigmentation. We found that hUMSCs‐Exos prominently reduced the infiltration of CD8^+^ T cells and enhanced the survival of melanocytes in mouse skin, with 3D‐Exos group displaying a better effect than 2D‐Exos group. Moreover, 3D‐Exos exhibited a superior effect in increasing the proportion of Treg cells in mouse tail skin and blood compared to 2D‐Exos group. Therefore, 3D‐Exos significantly inhibited the depigmentation in vitiligo mice by simultaneously promoting the expansion of Treg cells and reducing infiltrated CD8^+^T cells.

Based on the remarkable treatment effect of 3D‐Exos in pre‐clinical vitiligo mice model, exosome‐mediated immunomodulatory and anti‐oxidative effects and the underlying mechanisms were further investigated in vitro. After treated with hUMSCs‐Exos, the proportions of Treg cells in PBMCs and CD4^+^T cells were significantly increased and the effect of 3D‐Exos was more obvious, which is consistent with previous reports that MSCs‐derived exosomes can suppress pathological T cell subset activation and increase the abundance of Treg cells.^[^ ^42^ ^]^ Since that ROS can directly induce melanocyte apoptosis and also initiate autoimmune response to trigger melanocyte immune destruction in vitiligo,^[^ ^25^, ^43^ ^]^ we therefore observed the anti‐oxidative and anti‐apoptotic effect of hUMSCs‐Exos on melanocytes under H_2_O_2_‐induced oxidative stress in vitro. Compared with PBS treatment group, hUMSCs‐Exos pre‐treatment group reduced the toxicity of H_2_O_2_ to vitiligo melanocytes as revealed by the down‐regulation of ROS production and apoptosis rate, and the effect of 3D‐Exos group was superior to 2D‐Exos group. Similar results have been observed in several studies showing that MSCs‐Exos protects nerve cells, cardiomyocytes and keratinocytes by reducing ROS and apoptosis levels in response to various types of oxidative stress.^[^ ^44^ ^]^ Collectively, in addition to immune‐modulatory effect via the expansion of Treg cells, 3D‐Exos also had a direct protective effect on vitiligo melanocytes under oxidative stress, which was better than 2D‐Exos.

Accumulative evidence has revealed that hUMSCs‐Exos exert their biological functions on target cells via the delivery of specific cargos, among which miRNAs are of great significance.^[^ ^45^ ^]^ Therefore, high‐throughput microRNA sequencing (miRNA‐seq) was applied to compare the miRNA profiles of 3D‐Exos and 2D‐Exos and identify critical miRNAs contributing to the superior effect of 3D‐Exos. Of note, the differential hUMSCs‐Exos miRNA expression profile under 2D and 3D culture conditions forwardly supports the notion that culture microenvironment can lead to non‐physiological deviations in hUMSCs biology. Subsequently, through a series of gain‐of‐function and loss‐of function experiments, we indicated that miR‐125b‐5p exerted the favorable effects on H_2_O_2_‐induced oxidative damage in vitiligo melanocytes, whereas miR‐132‐3p dramatically promoted the differentiation of Treg cells in CD4^+^ T cells. Therefore, miR‐125b‐5p and miR‐132‐3p enriched in 3D‐Exos alleviated the oxidative injury in vitiligo melanocytes and promoted the differentiation of Treg cells, jointly exerting protective effect on melanocytes against immune and oxidative destruction in vitiligo. Furthermore, *Bak1* and *Sirt1* were identified as the direct targets of miR‐125b‐5p and miR‐132‐3p and mediated their effect on oxidative stress‐induced apoptosis and Treg cells expansion, respectively. It has previously been reported *Bak1* plays a pro‐apoptotic role as a direct target gene of miR‐125b‐5p.^[^ ^45b^ ^]^ Hypoxia‐induced MSCs‐Exos inhibits the expression of *Bak1* and tumor protein p53 through miR‐125b, to improve myocardial apoptosis and promote myocardial ischemia repair.^[^ ^46^ ^]^ The results above imply that miR‐125b‐5p in 3D‐Exos might reduce oxidative stress and apoptosis of vitiligo melanocytes by inhibiting *Bak1* expression. In addition, studies have revealed that inhibition of *Sirt1* expression can increase Foxp3 acetylation and Foxp3 mRNA production, thus promoting the production and function of Foxp3^+^ Treg cells.^[^ ^47^ ^]^ Of note, *Sirt1*‐induced differentiation of Treg cells has been investigated in numerous diseases. Zheng et al. reported miR‐155‐5p regulated Th17/Treg cells balance by targeting *Sirt1*, thereby alleviating chronic periodontitis.^[^ ^48^ ^]^ Similarly, targeting *Sirt1* mitigated Graft‐versus‐Host Disease development by inhibiting alloreactive T cell activation and promoting Tregs stability in mice.^[^ ^49^ ^]^ In this regard, 3D‐Exos‐derived miR‐132‐3p probably promoted the differentiation of Treg cells by inhibiting *Sirt1* expression in CD4^+^T cells. Therefore, miR‐125b‐5p and miR‐132‐3p might be alternative candidates with potential therapeutic value for vitiligo treatment.

Herein, the limitations of the present study should be informed. First, the specific mechanism by which 3D culture condition enhances the biological activity of hUMSCs remains elusive, which is necessary for providing credible evidence to support the clinical application of 3D culture method in treating vitiligo. Second, although our data has suggested that 3D‐Exos can efficiently ameliorate vitiligo via the delivery of miR‐125b‐5p and miR‐132‐3p, further investigation is needed for the identification of alternative substances that play the therapeutic roles in exosomes. Third, the present study has showed that 3D‐Exos can significantly inhibit the process of epidermal depigmentation in mice with vitiligo, but whether it can induce the re‐pigmentation of mice with vitiligo remains to be clarified. Last but not least, the clinical significance and therapeutic advances of MSC‐derived 3D exosomes need forward in‐depth investigation, especially in large‐scale multi‐center clinical research.

**Figure 11:**
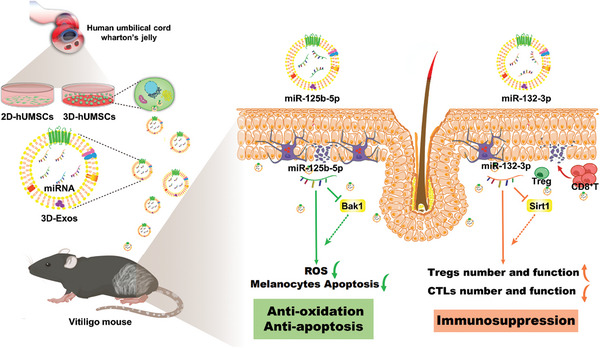
Schematic diagram of 3D‐Exos ameliorating vitiligo by potentiating Treg‐mediated immunesuppression and suppressing oxidative stress‐induced melanocyte damage.

Altogether, our study demonstrates that 3D culture condition can improve the production of hUMSCs‐Exos and enhance their anti‐oxidative and immune‐modulatory properties in vitro and in vivo compared with the traditional 2D culture condition. The 3D‐Exos harboring miR‐132‐3p and miR‐125b‐5p can induce Treg cells differentiation and suppress oxidative stress‐induced melanocyte apoptosis by targeting *Sirt1* and *Bak1* respectively, ultimately alleviating the progression of vitiligo (**Figure** **11**). Our present study intensifies the understanding of the therapeutic effect and the underlying mechanism of hUMSCs‐Exos in autoimmune disease, providing the proof of a principle that 3D‐Exos can be exploiting as a novel cell‐free therapy for a broad spectrum of autoimmune diseases due to its versatile biological functions.

In general, hUMSCs‐Exos could significantly suppress vitiligo mouse depigmentation in vivo. Mechanistically, the 3D‐Exos enriched with miR‐132‐3p and miR‐125b‐5p could induce Treg cells differentiation and suppress oxidative stress‐induced apoptosis by targeting *Sirt1* and *Bak1* respectively, thereby protecting melanocytes from oxidative and immune destruction, and ultimately alleviating the progression of vitiligo.

## 4 Experimental Section

### Isolation and Culture of hUMSCs

To collect hUMSCs, informed consent and ethics approval were obtained before all operations from all the donors. The research protocol was designed and executed according to the principles of the Declaration of Helsinki and was approved by the medical ethics committee of the first affiliated hospital of the Fourth Military Medical University (No. KY20213331‐1). hUMSCs were derived from Wharton's Jelly of umbilical cord tissue of selected healthy full‐term fetuses. In brief, the umbilical cord tissue was washed with sterile PBS, disinfected using 75% alcohol for 3 min, and cut into small segments. Followed by separating arteries and vein from the umbilical cord, the residual tissues were obtained and then cut into pieces, and placed in the cell incubator at 37 °C with 5% CO_2_. After about two weeks, hUMSCs can be seen to migrate from the tissues. hUMSCs at Passages 3–6 were used in the experiments. hUMSCs were cultivated in serum‐free MSCBM (DAKEWE, Shenzhen, China) containing EliteGro‐Adv (Elitecell, DAKEWE) mixed with 0.1% penicillin‐streptomycin (Sigma, St Louis, MO, USA). For 3D culture of hUMSCs, hUMSCs were digested when the confluent reached 80%−90%. The optimal cell density was 2×105 per mL, and the cell suspension (2 mL) was inoculated into ultra‐low adhesion culture 6‐well plate (CLS3471, Corning, NY, USA).

### hUMSCs‐Exos Isolation and Identification

Exosomes were obtained and enriched from supernatant collected from 2D‐hUMSCs and 3D‐hUMSCs by a series of ultracentrifugation as follows: Dead cells were abandoned at 800 g for 10 min, followed by cell fragments removed at 2000 g for 20 min. Subsequently, large extracellular vesicles were discarded at 20 000 g for 30 min, and exosomes were extracted at 100 000 g for 120 min and then filtered through a 0.22 µm filter. Furthermore, to dispose of impure residual proteins, the exosome pellet was rinsed with phosphate‐buffered saline (PBS), and the ultracentrifugation (100 000×g, 120 min) was repeated, after which exosomes were resuspended in PBS solution (200 µL) and stored at −80  °C. All procedures were conducted under 4 °C. For further identification, exosome morphology was visualized using TEM (Hitachi, Tokyo, Japan). Exosome suspension (10 µL) was dropped onto a copper grid, stained with 2% uranyl acetate solution for 1 min, and then images were obtained using TEM. The size and concentration of EVs were measured using the NanoFCM (NanoFCM Inc, Xiamen, China) as previously described.^[^ ^50^ ^]^ The PDI of exosome was measured using Zetasizer Nano‐S90 (Malvern Company, UK) and was carried out by Nuohai Life Science (shanghai) Co,td.

### Detection of Exosome Uptake by PIG3V Cells and PBMCs

To examine the take up of exosome by PIG3V cells and PBMCs, fluorescent labeling of 2D‐Exos and 3D‐Exos was carried out according to the manufacturer's instructions. Briefly, PKH26 dye working solution (100 µL, Umibio, Shanghai, China) was added to exosomes (100 µg). After maintaining at room temperature for 10 min, PBS (10 mL) was added for dilution. The excess dyes from labeled exosomes were removed by ultracentrifugation at 100 000×g for 120 min at 4 °C, and the stained exosomes were re‐suspended with PBS (200 µL). The PKH26‐labeled exosomes were co‐cultured with PIG3V cells and PBMCs for 24 h, and the cells were washed with PBS and fixed with 4% paraformaldehyde for 10 min, and permeated with 0.1% TritonX 100 for 10 min. Finally, cells were incubated with Fluor‐488‐conjugated Phalloidin (AAT Bioquest, CA, USA) for 90 min to stain F‐actin, and nuclear was stained with Hoechst 33 258 (Biyuntian Biotechnology, Shanghai, China). The uptake of PKH26‐labeled exosome by PIG3V cells and PBMCs was then observed by confocal laser scanning microscopy (Zeiss LSM 880). The fluorescence intensity of PKH26 was measured by image J software. PIG3V cells were cultured in DMEM/F12 medium supplemented with PMA (10 ng mL^−1^, HY‐18739, MCE, Beijing, China), CHO (200 nmol mL^−1^, Sigma, St Louis, MO, USA), bFGF (10 ng mL^−1^, Sino, Beijing, China) and 5% fetal bovine serum (FBS) (Gibico, Grand Island, NY). PBMCs were cultured in RPMI 1640 medium supplemented with 10% fetal bovine serum.

### Vector Constructs, Lentivirus Production, and Cell Transfections

The LV3‐human‐miR‐125b‐5p mimic vector, the LV3‐human‐miR‐132‐3p mimic vector, the LV3‐human‐miR‐574‐5p mimic vector (miROE), the LV3‐human‐miR‐125b‐5p‐inhibitor vector, and the LV3‐human‐miR‐132‐3p‐inhibitor vector (miRKD) were constructed by lentiviral vectors (GenePharma, Shanghai, China). A negative control was also constructed with the LV3 empty lentiviral (miR‐NCOE and miR‐NCKD). Upon reaching 40–50% confluency, the hUMSCs cultured in a 24‐well plate underwent virus infection based on the MOI values provided by the manufacturer. After cell proliferation, the cells underwent a 14‐day selection period with puromycin (1–4µg mL^−1^). The transfection effect was verified by RT‐PCR.

### Establishment of Vitiligo Mouse and Exosome Administration

Vitiligo mice model was establishment as previously described.^[^ ^19^ ^]^ Briefly, female C57BL6 mice at 8–9 weeks were injected intradermally with 2×105 B16F10 melanoma cells on day 0. To eliminate Tregs, anti‐CD4 mAb (10 µg g^−1^, clone GK1.5, Bio X Cell, USA) was administered by intraperitoneal injection on day 4 and 10. Primary tumors were surgically excised from the skin on day 12. Mice of vitiligo development were detected by harvesting tail ends for whole‐mount immunostaining on day 28. In subsequent studies, only mice with CD8^+^ T cell infiltration in the tail epidermis were used. Vitiligo mice were randomly divided groups, injecting PBS, 2D‐Exos (50 µg), 3D‐Exos (50 µg), 3D‐Exos^NCKD^ (50 µg), or 3D‐Exos^132KD^ (50 µg) weekly in the tail vein for 10 weeks respectively. Age‐ and sex‐matched C57BL6 wild‐type mice were used as sham group that received PBS intravenously only. Animal experiments were approved by the medical ethics committee of the first affiliated hospital of the Fourth Military Medical University (No. KY20213331‐1). Murine melanoma cell line B16F10 cells were purchased from American Type Culture Collection (American Type Culture Collection, Manassas, VA, USA). Specifically, the B16F10 cells growth medium was made of high‐glucose DMEM medium (Gibico, Grand Island, NY) supplemented with 10% fetal bovine serum (Gibco) and mixed with 0.1% penicillin‐streptomycin.

### Whole‐Mount Immunostaining

Whole‐mount immunostaining of mouse tail epidermis was performed as previously described.^[^ ^19^ ^]^ Briefly, the tail end of the mouse was cut off with the length of 1 cm and depilated with a depilatory cream. Skin tissue of mouse tail was obtained after removing mouse tail bone. Then the skin tissue of mouse tail was placed in ethylenediaminetetraacetic acid (EDTA) solution (20 mm) and incubated for 90 min at 37 °C with shaking. The epidermis and dermis were separated by fine tweezers. The isolated epidermis was fixed for 8 min in precooled 4% paraformaldehyde (PFA), washed with PBS, treated by 0.3% H_2_O_2_ in methanol solution at −20 °C for 20 min, and washed again with PBS. Afterward, sebaceous glands attached to the epidermis were removed under a stereomicroscope (M205 FA, Leica) and blocked for 5 h in BSA blocking solution containing 1% BSA, 0.3% TritonX‐100 and 2% Normal Donkey Serum. This was followed by incubation with primary antibodies for 12 h at 4 °C and secondary antibody for 8 h at 4 °C. After washed with PBS, the samples were sealed with 50% glycerol. The primary antibodies and secondary antibody are listed below: Rabbit monoclonal antibody to Melan‐A (1:500, Abcam, MA, USA), Rat monoclonal antibody to CD8α (1:300, Abcam), Cy3‐Donkey Anti‐Rat IgG (1:1000, Jackson ImmunoResearch, West Grove, PA, USA), Alexa Fluor 647‐Donkey Anti‐Rabbit IgG (1:1000, Jackson ImmunoResearch) and DAPI (1:1000, Dako, Glostrup, Denmark). Confocal fluorescence images were captured using confocal laser scanning microscopy (LSM 880, Carl Zeiss Microscopy, NY, USA), and data was analyzed using Imaris software (Bitplane, Oxford, UK) and R Studio to quantify the cell distribution and density as previously reported.^[^ ^19^ ^]^

### Flow Cytometry Analysis of Immune Cells in Vitiligo Mouse Tail Skin

To prepare the single cell suspension of mouse tail skin, the tail end of the mouse was cut off with the length of ≈3–4 cm, and then depilated and the tail bone was removed, as previously described in 4.6. The tail skin was cut into segments and incubated in Dispase solution (2.5 mg mL^−1^) at 37 °C for 30 min. Then the dermis and epidermis were separated. The dermis was put into collagenase IV solution (1 mg mL^−1^) and placed on a 37 °C shaker for digestion for 45 min. The epidermis was cut into pieces and placed on a 70 µm cell strainer and ground with a 5 mL syringe base. During this period, the single cell suspension from the epidermis was rinsed with PBS solution containing 1% FBS and deoxyribonuclease I (DNase I, 2 mg mL^−1^), filtered into a 15 mL centrifuge tube. After centrifugation at 1300 rpm for 8 min at 4 °C, the supernatant was discarded, and the precipitate was suspended again to obtain epidermal single cell suspension. Again, the dermis performs the same procedure to obtain dermal single‐cell suspension. The single‐cell suspension was collected, washed, and stained with FITC anti‐mouse CD45 (103 108, Biolegend), Percp anti‐mouse CD45 (103 130, Biolegend), APC‐Cy7 anti‐mouse CD8α (100 714, Biolegend), BV605 anti‐mouse CD69 (104 530, Biolegend), BV421 anti‐mouse CD25 (102 034, Biolegend), PE anti‐mouse CD4 (12‐0041‐81, eBioscience) for 30 min at room temperature. Then the cells were washed, fixed, permeabilized with the Fixation or Permeabilization Solution Kit (00‐5523‐00 Invitrogen), and stained intracellularly with PE anti‐mouse IFN‐γ (163 504, Biolegend) and FITC anti‐mouse Granzyme B (372 206, Biolegend). In addition, dead cells were stained according to the instructions using the Zombie Violet Fixable Viability Kit (423 113, BioLegend) or 7‐AAD vitality staining solution (420 403, BioLegend). Finally, these samples were analyzed by BD LSRFortessa flow cytometer (BD Biosciences). Data were analyzed with FlowJo software (Tree Star, Ashland, OR, USA).

### Flow Cytometry Analysis of Immune Cells in Spleen and Peripheral Blood in Vitiligo Mice

The single cell suspensions of spleen and peripheral blood were prepared as previously described.^[^ ^51^ ^]^ Briefly, the spleen was gently ground with the base of a 5 mL syringe until the spleen tissue became white and the spleen cell suspension was obtained through a 70 µm filter. Peripheral blood samples were collected by extracting the eyeball. Single cell suspensions of spleen and peripheral blood were centrifuged and followed by erythrocyte lysis with ammonium‐chloride‐potassium (ACK) buffer. Cells were washed twice with sterile PBS and enumerated. 1×106 cells were stained with fluorescent antibodies: FITC anti‐mouse CD4 (130 308, Biolegend), APC‐Cy7 anti‐mouse CD8α (100 714, Biolegend), BV605 anti‐mouse CD69 (104 530, Biolegend). After washing and centrifugation, the cells were fixed or permeabilized with the Fixation or Permeabilization Solution Kit (00‐5523‐00 Invitrogen) and then stained with intracellular molecules: PE anti‐mouse Foxp3 (320 008, Biolegend), PE‐Cy7 anti‐mouse IL‐10 (505 026, Biolegend), APC anti‐mouse TGF‐β(141 406, Biolegend), PE anti‐mouse IFN‐γ (163 504, Biolegend), FITC anti‐mouse Granzyme B (372 206, Biolegend). Finally, the samples were subjected to BD LSRFortessa flow cytometer (BD Biosciences). Data were analyzed with FlowJo software (Tree Star, Ashland, OR, USA).

### PBMCs, CD4^+^ T cells, and Naïve CD4^+^T Cells Isolation and Co‐Culture with hUMSCs‐Exos

PBMCs were isolated from both healthy donors and vitiligo patients' peripheral blood using Ficoll density gradient centrifugation. Approval for the research was granted by the medical ethics committee of the first affiliated hospital of the Fourth Military Medical University (Approval No. KY20213331‐1). CD4^+^T cells were extracted from PBMCs utilizing the MojoSort Human CD4^+^T cell Isolation Kit, while Naïve CD4^+^T cells were obtained using the Naive CD4^+^T Cell Isolation Kit II. These cells were suspended in RPMI 1640 supplemented with 10% fetal bovine serum and 0.1% penicillin‐streptomycin. Subsequently, PBMCs or CD4^+^T cells (5×105) were transferred into 24‐well plates and exposed to PBS, 2D‐Exos (50 µg mL^−1^), or 3D‐Exos (50 µg mL^−1^) for 3 days, followed by flow cytometry analysis. For gain‐ and loss‐of‐function studies, CD4^+^T cells (5×105) were treated with various exosomes (3D‐Exos^NCOE^, 3D‐Exos^125OE^, 3D‐Exos^132OE^, 3D‐Exos^574OE^, 3D‐Exos^NCKD^, 3D‐Exos^132KD^) at a concentration of 50 µg mL^−1^ for 3 days. Afterward, the cells were stimulated with CD3/CD28 activation magnetic beads for 24 hours and analyzed using a BD LSRFortessa flow cytometer.

In experiments concerning naïve CD4^+^T cell differentiation, cells (5×105) were co‐cultured with 2D‐Exos (50 µg mL^−1^) or 3D‐Exos (50 µg mL^−1^) in RPMI 1640 medium supplemented with IL‐2 (50 ng mL^−1^), TGF‐β (10 ng mL^−1^), and 10% fetal bovine serum. Following stimulation with CD3/CD28 activation magnetic beads for 5 days, changes in the proportion of Treg cells were evaluated.

To explore the secretion of immunosuppressive effector molecules from Treg cells, PBMCs (5×105) were co‐cultured with 2D‐Exos (50 µg mL^−1^) or 3D‐Exos (50 µg mL^−1^) in RPMI 1640 medium supplemented with 10% fetal bovine serum. The cells were then stimulated with CD3/CD28 activation magnetic beads for 48 h and Cell Activation Cocktail (with Brefeldin A) for 6 h before analysis using a BD LSRFortessa flow cytometer.

The cells were then stained with antibodies obtained from BioLegend: FITC anti‐human CD4 (300 506), BV605 anti‐human CD25 (302 632), APC anti‐human CD25 (302 610), BV421 anti‐human Foxp3 (320 124), PE anti‐human Foxp3 (320 008), PE‐Cy7 anti‐human IL‐10 (501 420), APC anti‐human TGF‐β (300 006).

### Treg and CD8^+^T Cell Coculture and Treg Cell Proliferation Assay

PBMCs were isolated from fresh blood samples of both healthy controls (HCs) and vitiligo patients using lymphocyte separation medium.CD8^+^T cells were extracted from PBMCs using the CD8^+^ T Cell Isolation Kit for human (Miltenyi Biotec, Bergisch Gladbach, Germany) as per the manufacturer's protocol. Tregs were then isolated from PBMCs using the CD4^+^CD25^+^CD127^dim/−^ Regulatory T Cell Isolation Kit (Miltenyi Biotec) following the manufacturer's instructions. For experiments involving co‐culture of Tregs and CD8^+^T cells, the specific procedure began with pre‐treating Treg cells with 3D‐Exos (50 µg mL^−1^) for 48 h. Subsequently, pre‐treated or untreated Treg cells (2.5×104 cells per well) were co‐cultured with CD8^+^T cells according to grouping. CD8^+^T cells were cultured in a 96‐well u‐bottom plate containing RPMI 1640 culture medium supplemented with 10% serum, with 1×105 cells per well, and activated with CD3/CD28 microbeads. For the CD8^+^T cell proliferation assay, cells were stained with CFSE (C34570, Invitrogen) and co‐cultured for 5 days. For the CD8^+^T cell activation assay, co‐culture was maintained for 24 h. For Treg cell proliferation experiments, following the same procedure as described above, Treg cells were stained with CFSE and co‐cultured for 5 days with 2D‐Exos (50 µg mL^−1^), 3D‐Exos (50 µg mL^−1^) and activating CD3/CD28 microbeads according to experimental conditions. Cell proliferation was assessed using flow cytometry based on the CFSE signal (FACS Calibur, BD, NJ, USA). The cells were then stained with antibodies obtained from BioLegend: FITC anti‐human CD4 (300 506), BV605 anti‐human CD25 (302 632), BV421 anti‐human Foxp3 (320 124), APC anti‐human CD69 (310 910).

### CCK8 Assay

PIG3V cells were seeded into 96‐well plates at a density of 5 × 103 cells per well, and incubated with different concentration of 2D‐Exos or 3D‐Exos (5, 10, or 20 µg mL^−1^) for 48 h. Moreover, PIG3V cells with 60%–80% confluence were treated with different doses of H_2_O_2_ (200–1000 µm) for 24 h. Then, each well was renewed with a mixture that contained of Cell Counting Kit‐8 detection working solution(10 µL) and cell culture medium(100 µL). After extra incubation for 1–2 h at 37 °C, the absorbance values were recorded with a microplate reader (Thermo Fisher Scientific, MA, USA) at 450 nm.

### MiRNA Library Preparation and Sequencing

The miRNA library preparation and sequencing of 2D‐Exos and 3D‐Exos were carried out by Hangzhou Lian chuan Biotechnology Co, Ltd. (https://www.omicstudio.cn/index). Briefly, total RNAs were extracted from exosomes (200 µg) purified from 2D‐hUMSCs and 3D‐hUMSCs supernatants, and both 2D‐Exos and 3D‐Exos groups contained 4 samples. TruSeq Small RNA Sample Prep Kits (Illumina, San Diego, USA) were used to prepare miRNA sequencing libraries. After the library was prepared, miRNA‐seq libraries were sequenced with a1×50 bp strand‐specific protocol on Illumina Hiseq 2000/2500. Sequencing data is accessible through GEO series accession numbers GSE211008.

### In Vivo Biodistribution of hUMSCs‐Exos by Fluorescence Optical Imaging

To examine the the organ biodistribution of hUMSCs‐Exos in vitiligo mice, fluorescent labeling of 2D‐Exos and 3D‐Exos was carried out according to the manufacturer's instructions. Briefly, DiR dye working solution (100 µL, Umibio, Shanghai, China) was added to exosomes (1000 µL). After maintaining at 37 °C for 30 min, PBS (10 mL) was added for dilution. The excess dyes from labeled exosomes were removed by ultracentrifugation at 100 000×g for 120 min at 4 °C, and the stained exosomes were re‐suspended with PBS (200 µL). The experiment was performed in vitiligo mice, randomly divided into groups. Freshly purified DiR‐labelled 2D‐Exos (50 µL) and 3D‐Exos (50 µL) were injected intravenously via the tail vein. Isoflurane‐sedated live mice were imaged using the IVIS Lumina XRMS Series 2 (PerkinElmer, Thermo Fisher, US) at 24 h following intravenous injection. The fluorescence intensity in each mouse (total radiant efficiency) was obtained using Living Image Software (PerkinElmer) to determine the organ biodistribution of DiR‐labelled 2D‐Exos and 3D‐Exos.

### Reactive Oxygen Species (ROS) Examination

The intracellular ROS of PIG3V cells was measured using a ROS assay kit (Beibo, Shanghai, China). Briefly, PIG3V cells were pre‐treated for 48 h with or without hUMSCs‐Exos or 3D‐Exo^miROE^ or 3D‐Exo^miRKD^ and then stimulated with H_2_O_2_ for 24 h. After the supernatant was discarded, the cells were washed twice with PBS, and then incubated with fluorescent probe DHE (PE‐Texas‐Red) in the dark at 37 °C for 30 min. The unbound probes were rinsed with PBS, and then the intracellular ROS was measured with a confocal laser scanning microscopy (Carl Zeiss Microscopy, NY, USA) or examined by BD LSRFortessa flow cytometer (BD Biosciences).

### Flow Cytometry Analysis of Cell Apoptosis

Apoptosis of PIG3V cells was performed using the apoptosis kit (Seven Sea PharmTech, Shanghai, China) according to the manufacturer's instructions. Briefly, PIG3V cells were pretreated for 48 h with or without hUMSCs‐Exos or 3D‐Exo^miROE^ or 3D‐Exo^miRKD^ and then stimulated with H_2_O_2_. After treatment, cells in supernatant and culture plates were collected and then washed twice with PBS. Apoptotic cells were stained with annexin V(10 µL) and propidium iodide(5 µL), respectively, and then analyzed by BD LSRFortessa flow cytometer (BD Biosciences). Cell apoptosis rate was the ratio of early apoptosis cells and advanced apoptosis cells to total number of cells.

### Dual‐Luciferase Reporter Assay

Luciferase reporter transfection and dual‐luciferase assays were performed as described previously.^[^ ^52^ ^]^ The reporter vectors of *Bak1* and *Sirt1* containing wild type and mutant miR‐125b‐5p and miR‐132‐3p binding sites were constructed separately and then cloned into psiCHECKTM‐2 Vector (Promega, Madison, WI, USA). 293T cells were seeded in 24‐well plates and incubated for 24 h before transfection. Next, the luciferase vector (0.5 µg) was co‐transfected with indicated miRNA using lipofectamine 2000 (Invitrogen). After transfecting for 48 h, the cells Renilla and firefly luciferase activities were measured using dual‐luciferase reporter assay system (Promega, Madison, WI, USA).

### RNA Isolation and Reverse‐Transcription Quantitative RT‐PCR

Total RNA was isolated and extracted from exosomes and the cells including hUMSCs, PIG3V cells, and CD4^+^ T cells after application of Trizol reagent (Invitrogen). The RNA was reversely transcribed to cDNA by employing the PrimeScript RT Reagent Kit (TaKaRa, Tokyo, Japan). qRT‐PCR was further performed to estimate the levels of *Bak1* and *Sirt1* by the SYBR Premix Ex Taq II Kit (TaKaRa). β‐actin was set as an internal standard. For the miRNAs expression detection, RNAs were reversely transcribed using the miRcute miRNA first‐strand cDNA synthesis kit (Tiangen, Beijing, China). qRT‐PCR reactions were performed to estimate the expression levels of miR‐132‐3p, miR‐125b‐5p, miR‐574‐5p, miR‐21‐5p, and miR‐100‐5p using the miRcute miRNA qPCR detection kit (Tiangen). U6 was set as an internal standard. In the verification of miRNAs, reverse primer was the universal primer in the kit, and miRNAs‐specific forward primers for miR‐132b‐3p, miR‐125b‐5p, miR‐574‐5p, miR‐21‐5p, miR‐100‐5p, and U6 were purchased from RiboBio Co. Ltd. The relative mRNA or miRNA expression was calculated using the 2^−△△Ct^ method. The primer sequences were listed as following. *Bak1* forward: 5′‐ATGGTCACCTTACCTCTGCAA −3′; reverse: 5′‐TCATAGCGTCGGTTGATGTCG −3′; *Sirt1* forward: 5′‐ TAGAGCCTCACATGCAAGCTCTA −3′; reverse: 5′‐ GCCAATCATAAGATGTTGCTGAAC −3′; β‐actin forward: 5′‐GACAGTCAGCCGCATCTTCT‐3′; reverse: 5′‐GCGCCCAATACGACCAAATC‐3′.

### Western Blot Analysis

Radio immunoprecipitation assay (RIPA) buffer (Beyotime biotechnology, Shanghai, China) was added with protease inhibitor (Temekuramillipore, California, USA) for the lysis of cells. Subsequently, the lysates were centrifuged at 12 000 g for 15 min to extract protein. The concentration of total protein was detected using BCA protein analysis kit (Pierce, Rockford, Illinois, USA). The same quantity of protein was isolated by 10% sodium dodecyl sulfate‐poly acrylamide gel electrophoresis (SDS‐PAGE), and then transferred to nitrocellulose membranes (Millipore, Temecula, California, USA). Subsequently, the membrane was sealed with 5% defatted milk at 37 °C for 1 h, and incubated with primary antibody at 4 °C. After 10 h, the membrane was incubated with horseradish peroxidase (HRP)‐labeled secondary anti mouse IgG or anti rabbit IgG antibody (Abcam, Cambridge, Massachusetts, USA) at 37 °C for 1 h. The primary antibodies were used to detect protein expression as following: CD9 (1:1000, 60232‐1‐Ig, Proteintech, Wuhan, China), TSG101 (1:2000, 67381‐1‐Ig, Proteintech), Alix (1:2000, 12422‐1‐AP, Proteintech), *Bak1* (1:1000, #12 105, Cell signaling technology, Beverly, Massachusetts), *Sirt1* (1:1000, ab32441, Abcam, Cambridge, Massachusetts, USA), Fetuin A(1:1000, 16571‐1‐AP, Proteintech, Wuhan, China), ICAM1(1:1000, A5597, Abclonal, China), and β‐Actin (1:5000, #3700, Cell signaling technology). The protein bands were visualized by enhanced chemiluminescence detection system (Bio rad, California, USA) and analyzed by using Image J software.

### Statistical Analysis

At least three independent replicates were performed to ensure the accuracy of all experiments’ results. Sample size and data pre‐processing, including normalization, are detailed in the figure legends to provide transparency and context for the data presentation. Data are shown as mean ± standard deviation. Statistical analysis was carried out using GraphPad software 9.0 and SPSS 22.0. To calculate the *P* values, Student's *t* test was performed for two‐group comparisons, and one‐way ANOVA was utilized for more than two‐group comparison. *P* < 0.05 was set to be statistically significant (*p < 0.05, **p < 0.01, ***p < 0.001, ****p < 0.0001).

## Conflict of Interest

The authors declare no conflict of interest.

## Supporting information

Supporting Information

## Data Availability

The data that support the findings of this study are available from the corresponding author upon reasonable request.
