# Supplementary material for: 3D‐hUMSCs Exosomes Ameliorate Vitiligo by Simultaneously Potentiating Treg Cells‐Mediated Immunosuppression and Suppressing Oxidative Stress‐Induced Melanocyte Damage
Source: Adv Sci (Weinh). 2024 Jun 17;11(31):2404064. doi: 10.1002/advs.202404064 (PMC11336971; doi:10.1002/advs.202404064)
Supplement: Supplementary file 1 — Supporting Information [file ADVS-11-2404064-s001.docx]

Supporting Information

**3D-hUMSCs Exosomes Ameliorate Vitiligo by Simultaneously Potentiating Treg Cells-mediated Immunosuppression and Suppressing Oxidative Stress-induced Melanocyte Damage**

Qi Wang^1#^, Weinan Guo^1#^, Liaoran Niu^2^, Yuqi Zhou^1^, Zeqian Wang^1^, Jianru Chen^1^, Jiaxi Chen^1^, Jingjing Ma^1^, Jia Zhang^1^, Zhaoting Jiang^1^, Bo Wang^1^, Zhe Zhang^1^, Chunying Li^*^, Zhe Jian^*^

**^1^** **Department of Dermatology, Xijing Hospital, Fourth Military Medical University,** **Xi’an, Shaanxi 710032, China**

**^2^** **Department of Digestive Surgery, Xijing Hospital, Fourth Military Medical University, Xi’an, Shaanxi 710032, China**

**^#^ The authors contributed equally to the work.**

***Correspondence**

Zhe Jian, Department of Dermatology, Xijing Hospital, Fourth Military Medical University, Xi’an, Shaanxi 710032, China.

Email: [xjzhejian@fmmu.edu.cn](mailto:xjzhejian@fmmu.edu.cn)

Chunying Li, Department of Dermatology, Xijing Hospital, Fourth Military Medical University, Xi’an, Shaanxi 710032, China.

Email: [lichying@fmmu.edu.cn](mailto:lichying@fmmu.edu.cn)

**
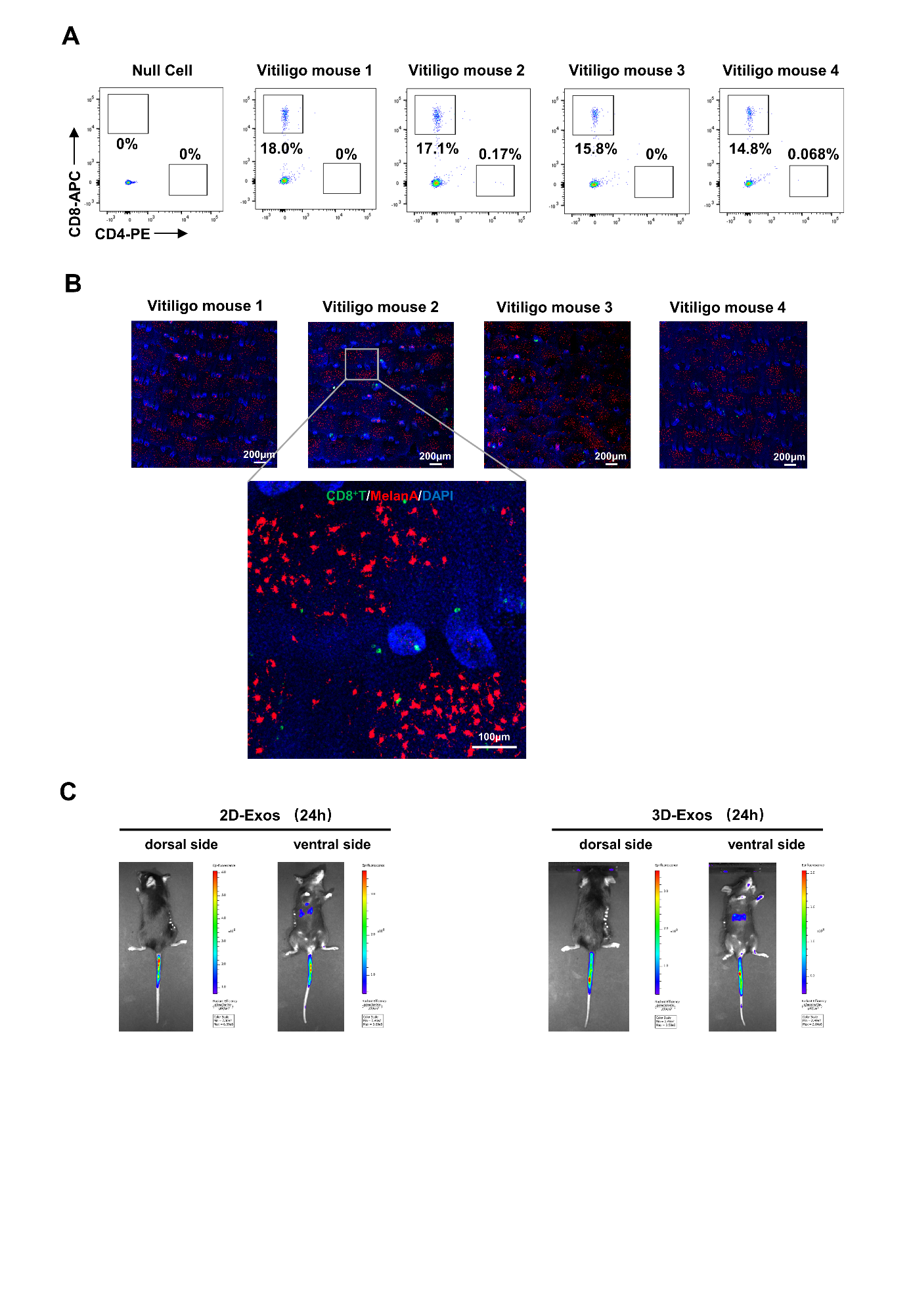
**

**Figure S1. Validation of vitiligo mouse model and *in vivo* imaging after exosome injection.**

A) Flow cytometry was used to analyze the proportions of CD4^+^T and CD8^+^T cells in tail venous blood of mice after CD4 antibody injection. B) Whole-mount immunofluorescent staining images of CD8^+^T cells (green) and melanocytes (red) in the tail epidermis of vitiligo mice (*n*= 3 or 4 per group). C) Whole-body imaging 24 h after intravenous injection of DIR-labeled 2D-Exos and 3D-Exos.

**
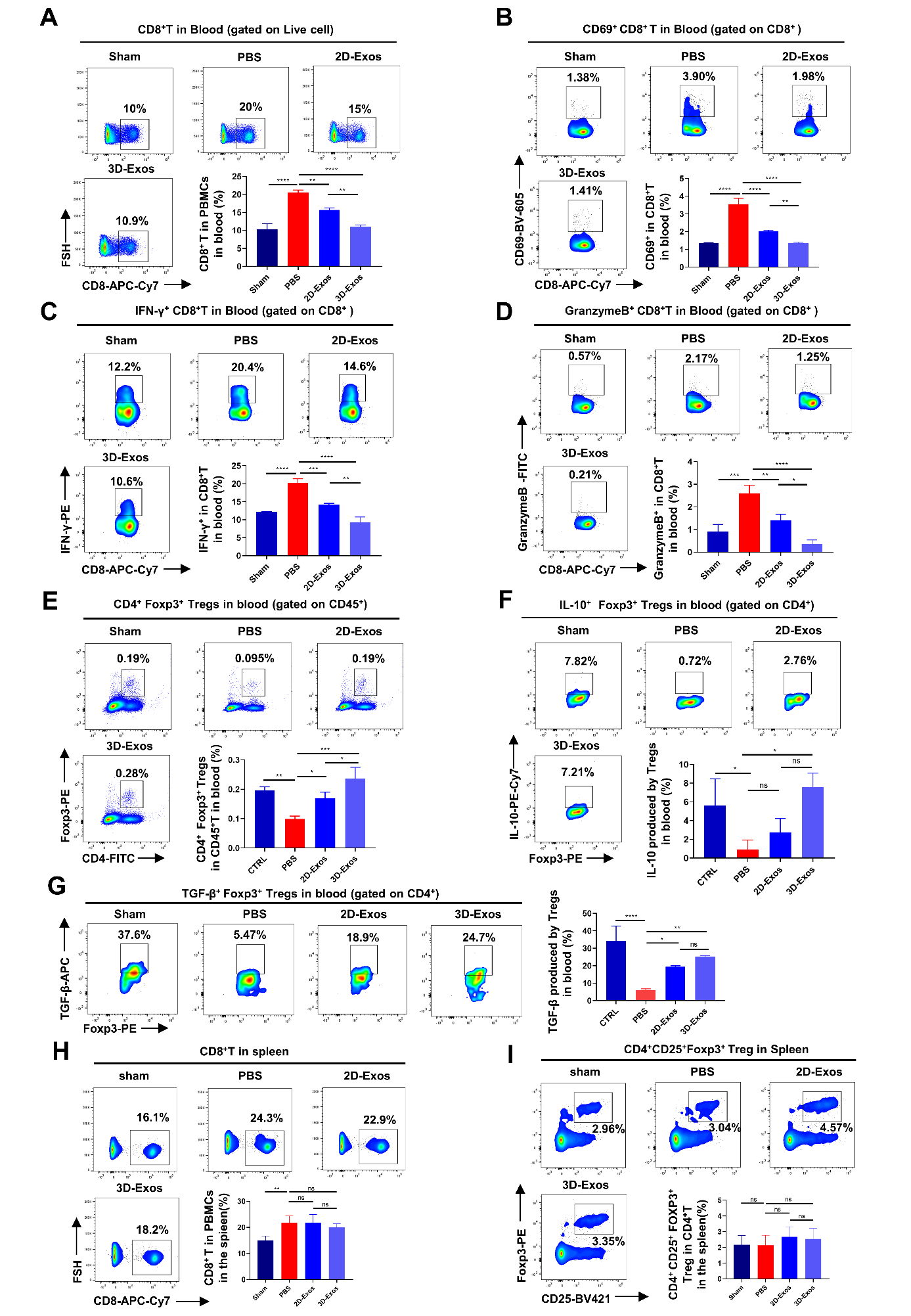
**

**Figure S2. Effect of hUMSCs-Exos on the CD8^+^T and Treg cells in the blood and spleen**

**of vitiligo mice.**

1. Flow cytometry and statistical analysis of the effect of PBS, 2D-Exos or 3D-Exos on

CD8^+^T cells proportions in blood (*n*= 3 per group). B-D) Flow cytometry and statistical analysis of the effects of PBS, 2D-Exos or 3D-Exos on the expression levels of the activation marker CD69, IFN-γ and GranzymeB in CD8^+^T cells in blood (*n*= 3 per group). E) Flow cytometry and statistical analysis of the effect of PBS, 2D-Exos or 3D-Exos on Treg cells proportions in blood (*n*= 3 per group). F,G) Flow cytometry and statistical analysis of the effect of PBS, 2D-Exos or 3D-Exos on the expression of IL-10 and TGF-β in Treg cells in blood (*n*= 3 per group). H,I) Flow cytometry and statistical analysis of the effect of PBS, 2D-Exos or 3D-Exos on CD8^+^T cells and Treg cells proportions in spleen (*n*= 3 or 4 per group). All sham mice were only treated with PBS (*n*= 3 per group). Error bars represented mean ± SD. Statistical significance was denoted as ^*^*p* < 0.05, ^**^*p* < 0.01, ^***^*p* < 0.001 and ^****^*p* < 0.0001 by one way analysis of variance (ANOVA) and Tukey’s test; ns indicates not statistically significant.


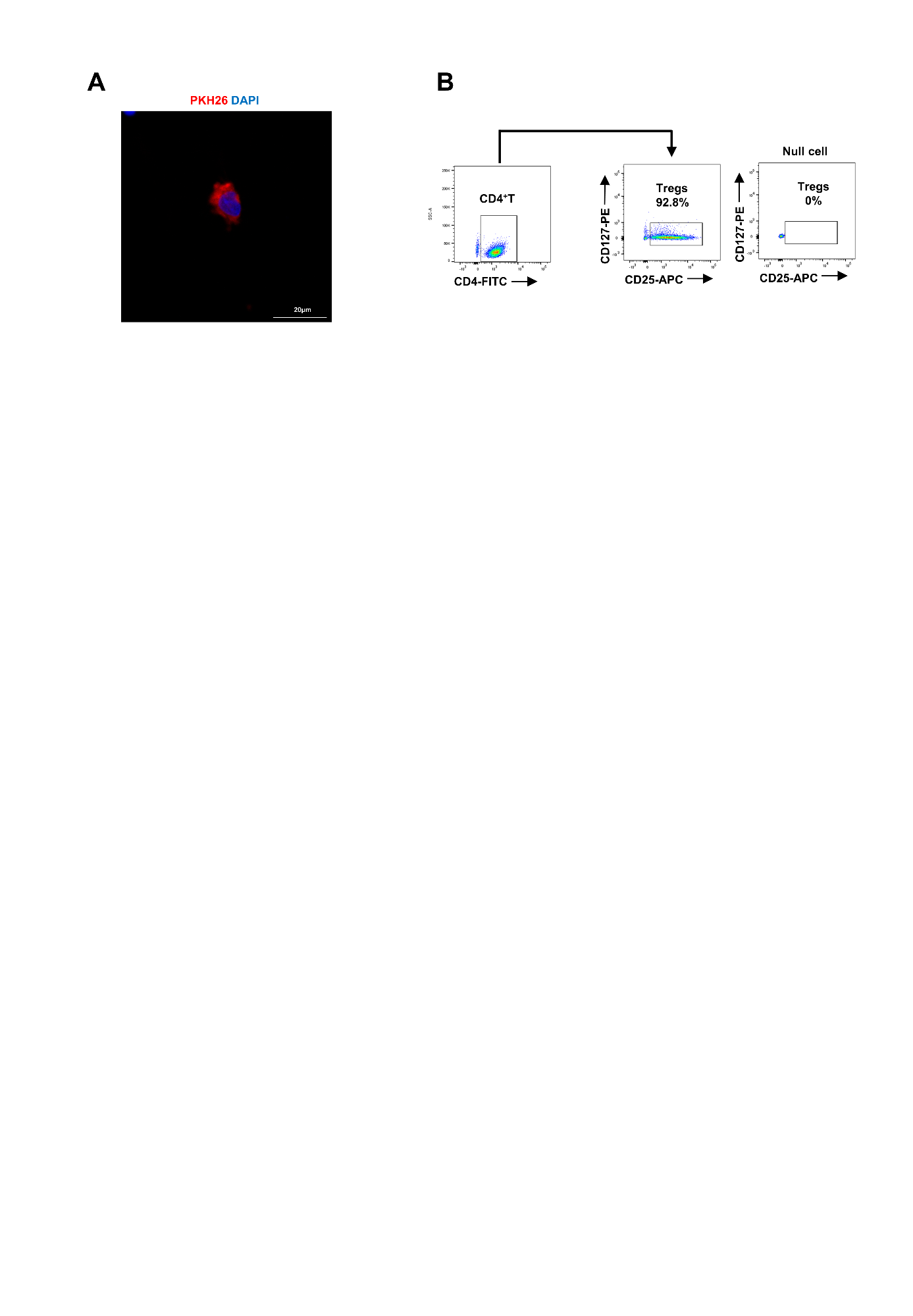


**Figure S3.** **PBMCs were incubated with PKH26-labeled 3D-Exos, followed by flow**

**cytometry analysis to assess the purity of Treg cells.**

1. Uptake of the PKH26-labeled 3D-Exos by PBMC was observed by fluorescence

microscopy. B) Flow cytometry to detect the purity of Treg cells.

**
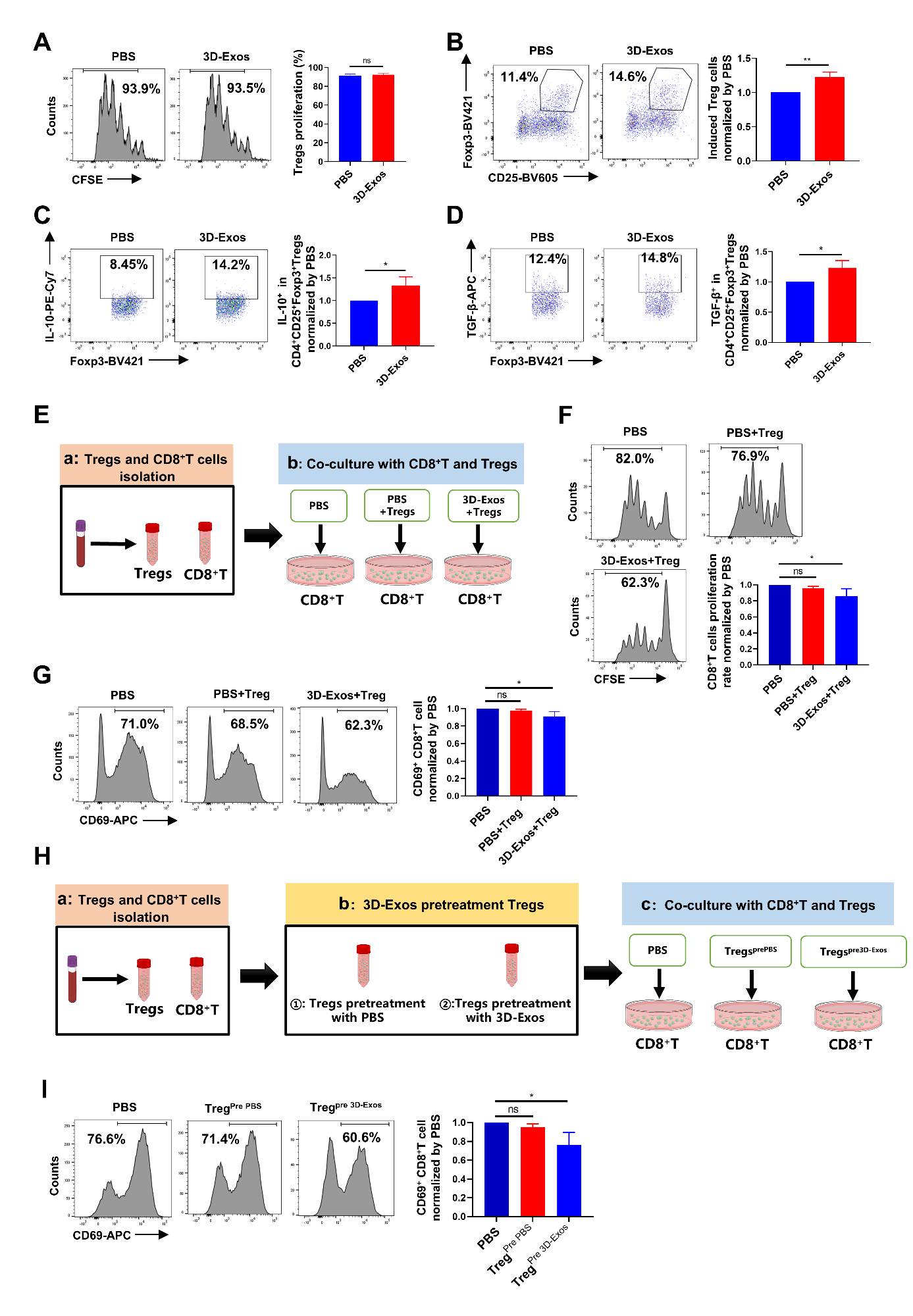
**

**Figure S4. 3D-Exos treatment could restore the suppressive function of Treg cells in the**

**peripheral blood of vitiligo patients in vitro.**

1. CFSE-labeled Tregs were cultured with or without 3D-Exos for 5 days in the presence of

CD3/CD28 microbeads. The percentage of proliferating cells was assessed by flow cytometry (*n*= 4 per group). B) Naive CD4^+^T cells were co-cultured with or without 3D-Exos in medium containing IL-2 and TGF-β. Flow cytometry was performed to detect the proportion of Treg cells after 5 days of stimulation with CD3/CD28 beads, with all groups normalized to the PBS group (*n*= 3 per group). C,D) PBMC were cultured with or without 3D-Exos for 48h in the presence of CD3/CD28 microbeads. The percentage of IL-10 and TGF-β expression in Treg was assessed by flow cytometry, with all groups normalized to the PBS group (*n*= 4 per group). E) Diagram of co-culture of Treg cells and CD8^+^T cells. F) CFSE-labeled CD8^+^T cells were cultured with or without 3D-Exos or Tregs for 5 days in the presence of CD3/CD28 microbeads. The percentage of proliferating cells was assessed by flow cytometry, with all groups normalized to the PBS group (*n*= 3 per group). G) The CD8^+^ T cells were co-cultured with or without Treg or 3D-Exos for 24 h in the presence of CD3/CD28 microbeads, and the expression levels of CD69 were assessed by flow cytometry, with all groups normalized to the PBS group (*n*= 4 per group). H) The diagram illustrated the co-culture of Treg cells with and without 3D-Exos pre-treatment, along with CD8^+^T cells. I) The Treg cells, pre-treated with or without 3D-Exos, were co-cultured with CD8^+^T cells for 24 h in the presence of CD3/CD28 microbeads, and the expression levels of CD69 were assessed by flow cytometry. with all groups normalized to the PBS group (*n*= 3 per group). Error bars represented mean ± SD. Statistical significance was denoted as ^*^*p* < 0.05, ^**^*p* < 0.001 by one-way analysis of variance (ANOVA) and Tukey’s test (F, G, I) or by t test (A, B, C, D); ns indicates not statistically significant.

**
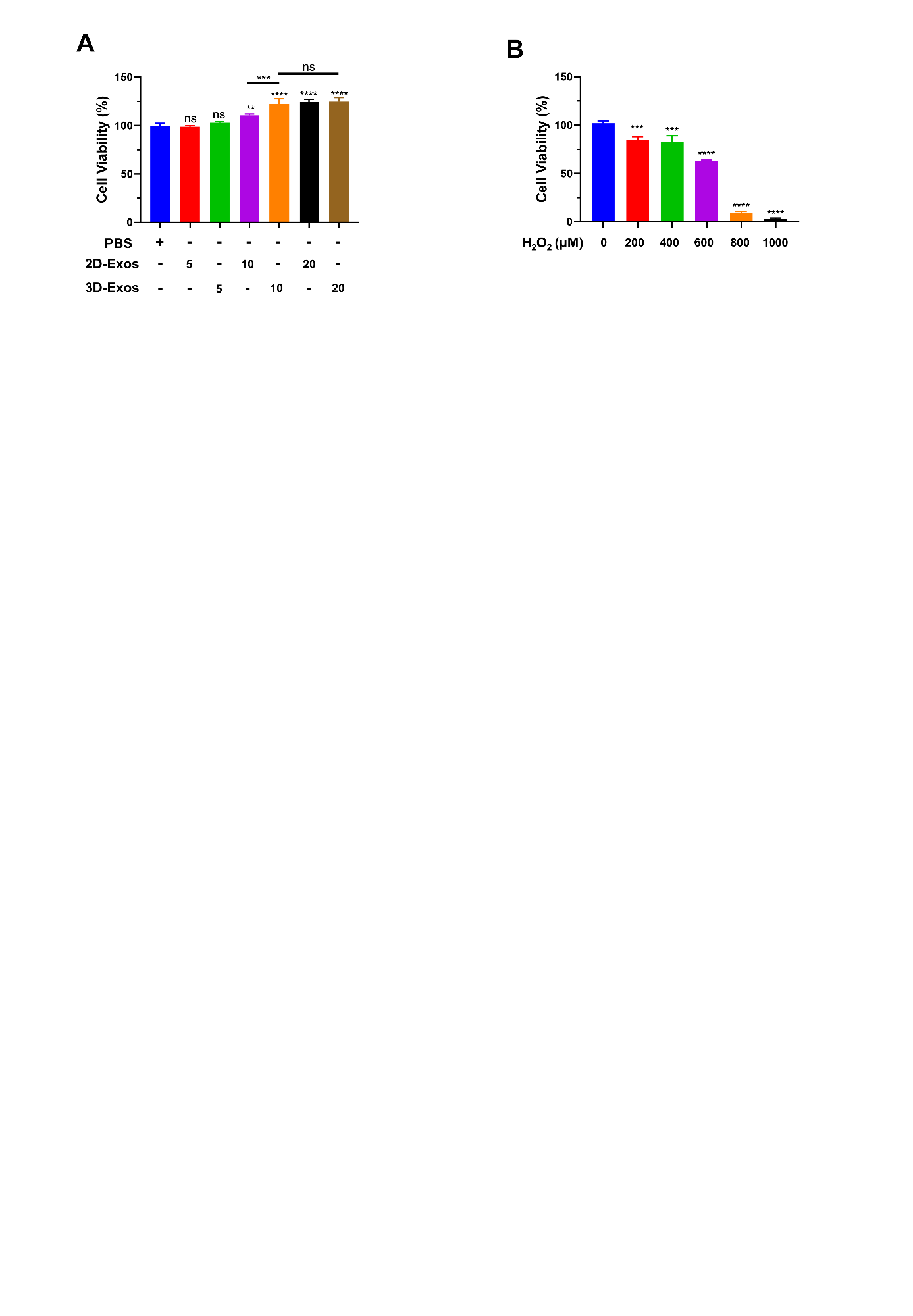
**

**Figure S5. Co-culture of hUMSCs-Exos with PIG3V cells and establishment of H_2_O_2_-induced oxidative stress model.**

A) Cell viability of PIG3V cells after the co-culture with hUMSCs-Exos (*n*= 4 per group). B) PIG3V cells treated with increasing doses of H_2_O_2_, and the cell viability was detected using a CCK8 assay (*n*= 3 per group). Error bars represented mean ± SD. Statistical significance was denoted as ^**^*p* < 0.01, ^***^*p* < 0.001, and ^****^*p* < 0.0001 by one-way analysis of variance (ANOVA) and Tukey’s test (A, B); ns indicates not statistically significant.

**
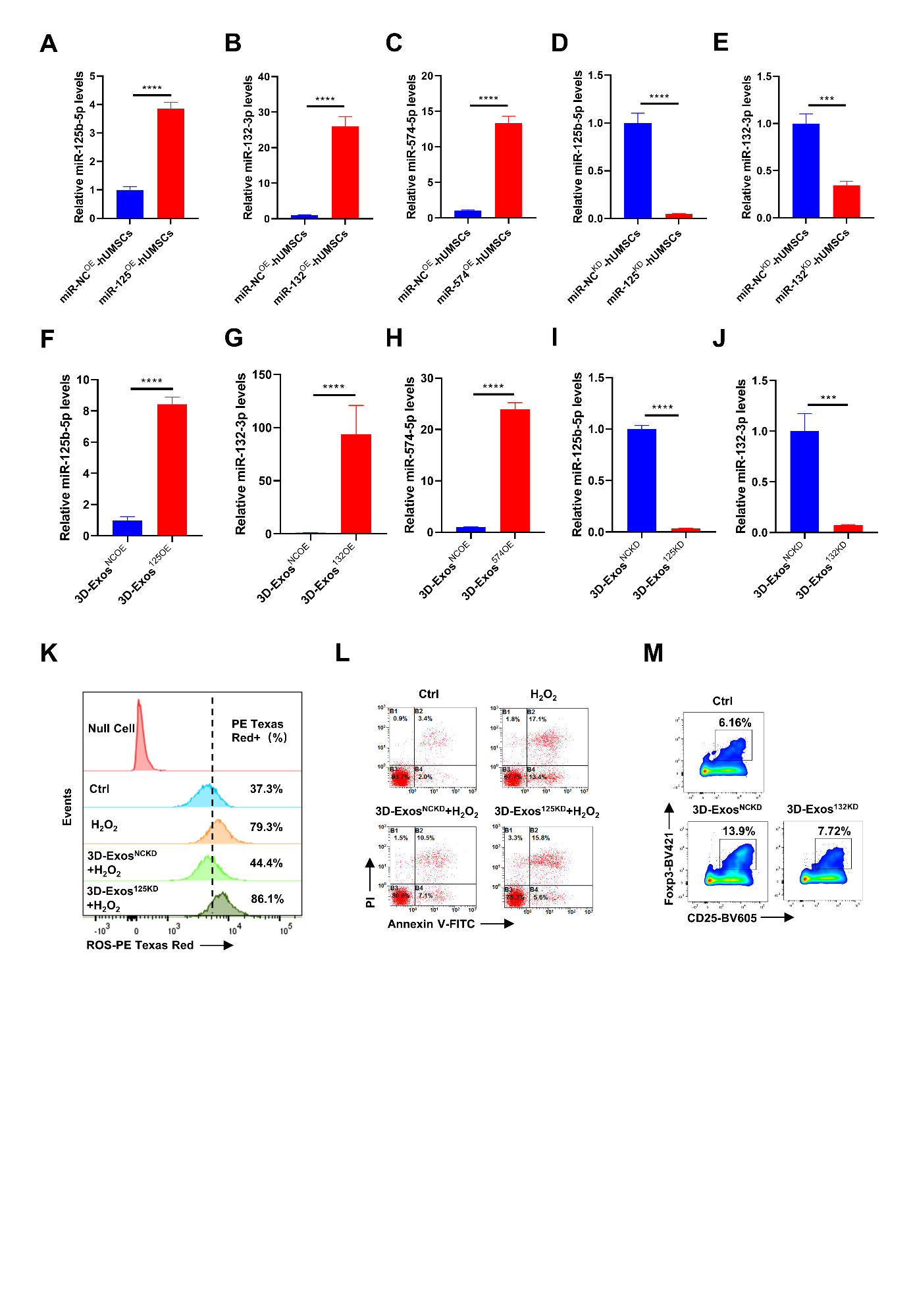
**

**Figure S6. The establishment of hUMSCs with the overexpression or knockdown of specific miRNA, and subsequent flow cytometry analysis associated with loss-of-function studies.**

A-E) miR-125b-5p, miR-132-3p, miR-574-5p overexpression and miR-125b-5p, miR-132-3p knockdown in hUMSCs and the efficiency confirmed using qRT-PCR. F-J) The relative expression level of miR-125b-5p, miR-132-3p, miR-574-5p in exosomes derived from 3D cultured hUMSCs transfected with the LV3-human-miR-125b-5p/miR-132-3p/miR-574-5p mimic vector and the LV3-human-miR-125b-5p/ miR-132-3p inhibitor vector. K) Effects of 3D-Exos^NCKD^ or 3D-Exos^125KD^ on intracellular ROS levels in PIG3V cells by flow cytometry analysis (*n*=4 per group). L) Effects of 3D-Exos^NCKD^ or 3D-Exos^125KD^ on cell apoptosis of PIG3V cells by flow cytometry analysis (*n*=3 per group). M) Effect of 3D-Exos^NCKD^ or 3D-Exos^132KD^ on proportion of Treg cells in CD4^+^T cells(*n*=3 per group). Error bars represented mean ± SD. Statistical significance was denoted as ^***^*p* < 0.001, ^****^*p* < 0.0001 by t test (A-J).

**
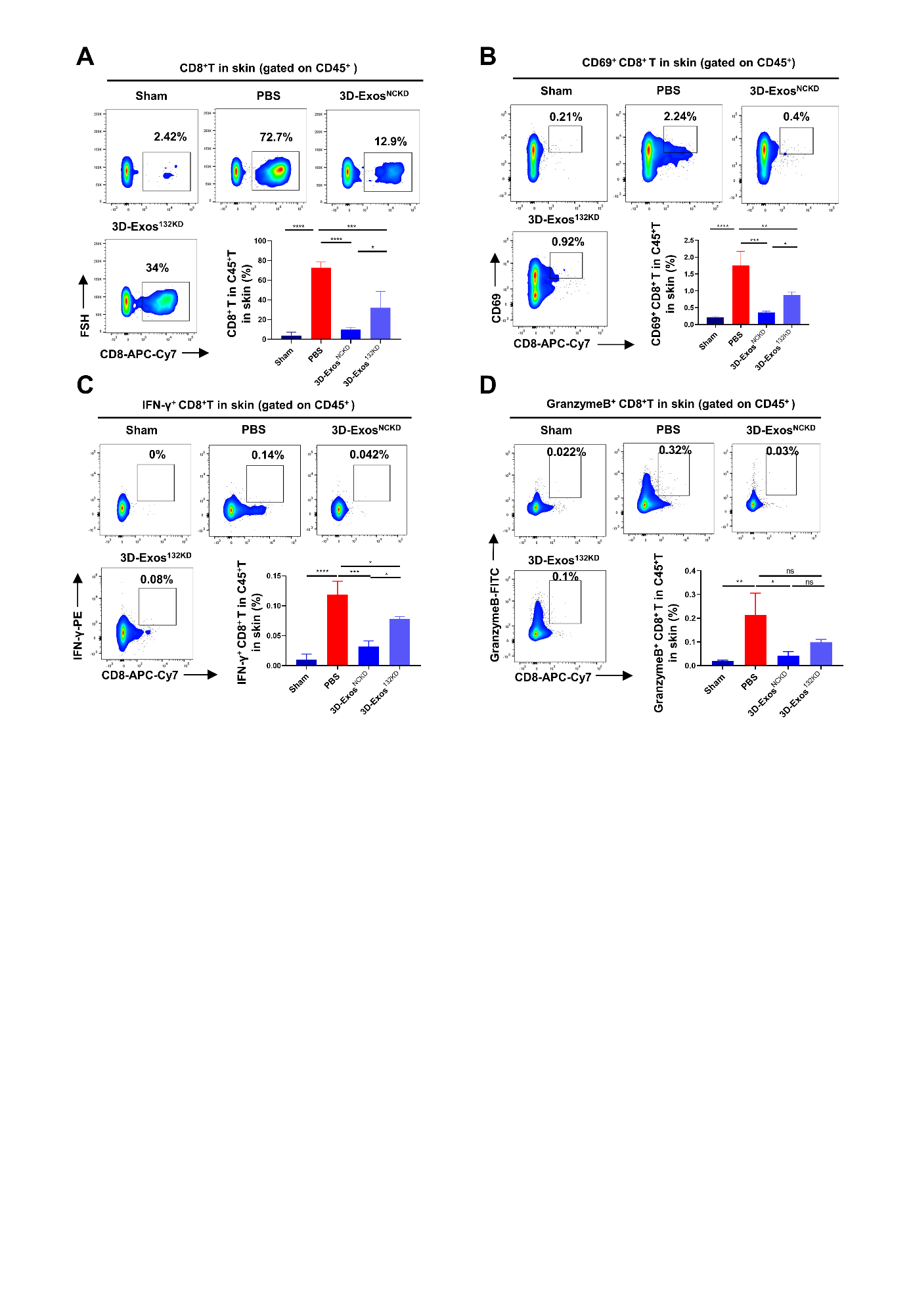
**

**Figure S7. Effect of 3D-Exos^132KD^ on the CD8^+^T in the skin of vitiligo mice.**

1. Flow cytometry and statistical analysis of the effect of PBS, 3D-Exos^NCKD^ or

3D-Exos^132KD^ on CD8^+^T cells proportions in skin (*n*= 3 per group). B-D) Flow cytometry and statistical analysis of the effects of PBS, 3D-Exos^NCKD^ or 3D-Exos^132KD^ on the expression levels of the activation marker CD69, IFN-γ and GranzymeB in CD8^+^T cells in skin (*n*= 3 per group). All sham mice were only treated with PBS (*n*= 3 or 4 per group). Error bars represent mean ± SD. Statistical significance was denoted as **p* < 0.05, ***p* < 0.01, ****p* < 0.001 and *****p* < 0.0001 by one way analysis of variance (ANOVA) and Tukey’s test; ns indicates not statistically significant.

**
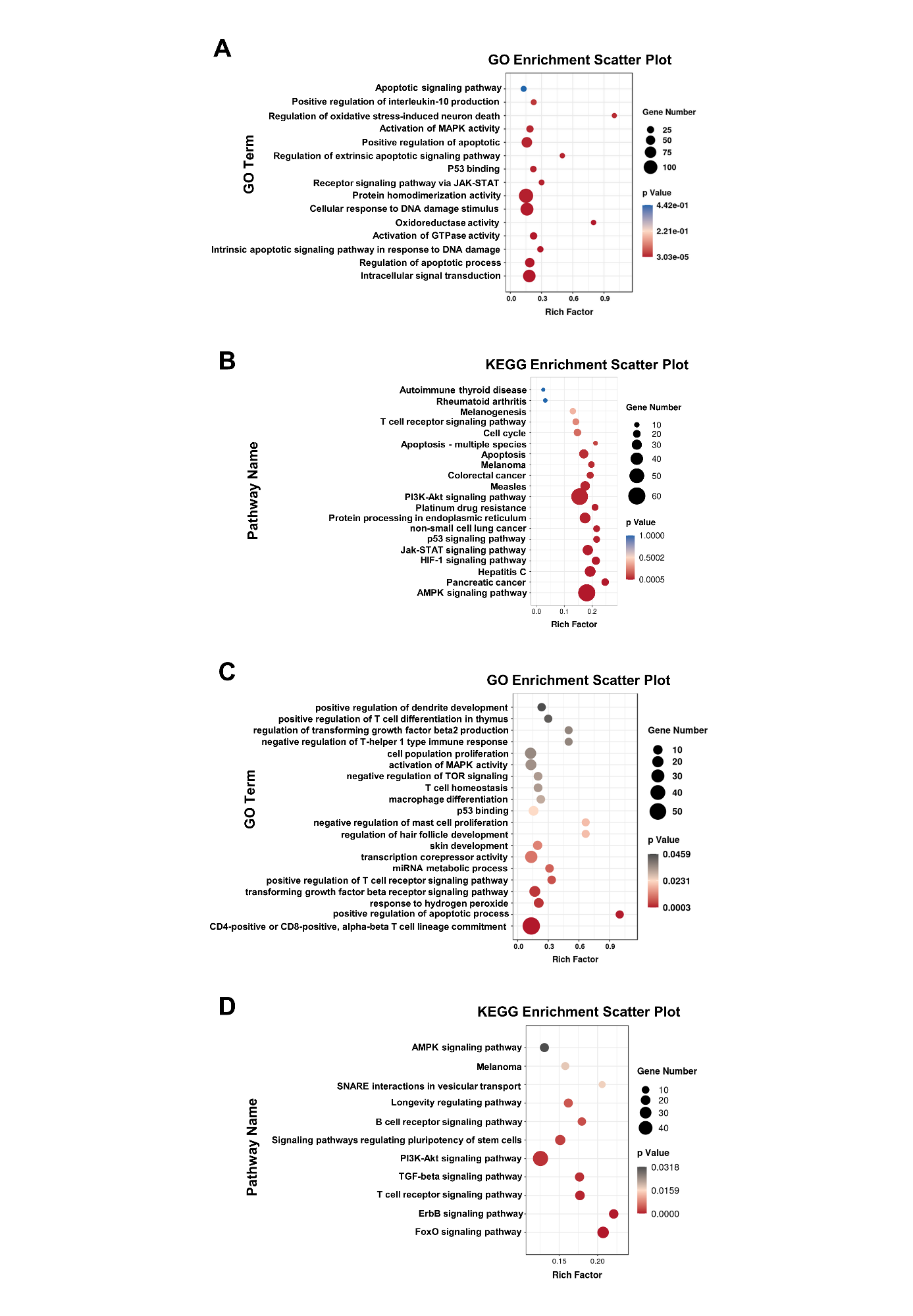
**

**Figure S8. Bioinformatics analyses of predicted target genes of miR-125b-5p and miR-132-3p.**

A,B) GO and KEGG analysis of predicted target genes of miR-125b-5p. C,D) GO and KEGG analysis of predicted target genes of miR-132-3p.
